# An Overview of the Use of Equine Collagen as Emerging Material for Biomedical Applications

**DOI:** 10.3390/jfb11040079

**Published:** 2020-11-01

**Authors:** Nunzia Gallo, Maria Lucia Natali, Alessandro Sannino, Luca Salvatore

**Affiliations:** Department of Engineering for Innovation, University of Salento, Via Monteroni, 73100 Lecce, Italy; marialucia.natali@unisalento.it (M.L.N.); alessandro.sannino@unisalento.it (A.S.); luca.salvatore@unisalento.it (L.S.)

**Keywords:** type I collagen, equine, biomaterials, medical devices

## Abstract

Type I collagen has always aroused great interest in the field of life-science and bioengineering, thanks to its favorable structural properties and bioactivity. For this reason, in the last five decades it has been widely studied and employed as biomaterial for the manufacture of implantable medical devices. Commonly used sources of collagen are represented by bovine and swine but their applications are limited because of the zoonosis transmission risks, the immune response and the religious constrains. Thus, type-I collagen isolated from horse tendon has recently gained increasing interest as an attractive alternative, so that, although bovine and porcine derived collagens still remain the most common ones, more and more companies started to bring to market a various range of equine collagen-based products. In this context, this work aims to overview the properties of equine collagen making it particularly appealing in medicine, cosmetics and pharmaceuticals, as well as its main biomedical applications and the currently approved equine collagen-based medical devices, focusing on experimental studies and clinical trials of the last 15 years. To the best of our knowledge, this is the first review focusing on the use of equine collagen, as well as on equine collagen-based marketed products for healthcare.

## 1. Introduction

Collagen is the body’s cement that keeps everything in place [1]. With its 28-members family it is the most important protein of vertebrates’ connective tissues that accounts for the 30% of the total body protein content [2]. Among collagens, fibril-forming type I subspecies is the most abundant since it accounts for the 70% of the whole family [3,4,5]. The structure of type I collagen, distributed at the level of all tissues in the organism, is known from 1938 [6,7,8]. It consists in three right-handed polyproline-II helices of about 1000 amino acids (called α strand) that by mean of interchain hydrogen bonds are held together in a left-handed triple helix [9]. As it is already known, each α strand is characterized by the repetition of the Gly-X-Y triplet, where the “X” and “Y” positions are usually occupied by proline and hydroxyproline [10]. In this neat sequence, glycine plays a key role in the three α strand packing [11], while proline and hydroxyproline cover a fundamental role in stabilizing the triple helical structure by preventing helices free rotation, thanks to the presence of pyrrolidine rings which reduce the degree of freedom of the polypeptide chain [12,13]. Moreover, the post-translational hydroxylation of the 11%–14% of proline residues by the enzyme proxyl-4-hydroxylase (PH4) (EC 1.14.11.2) is a process that gives to collagen a unique characteristic attributable only to type I collagen, important both for its recognition and quantification [14].

Collagen covers a crucial structural role for the maintenance of tissues’ architecture and shape and it dictates specialized regulatory functions, especially during development and repair [15,16,17]. Thus, collagen is not only responsible of tensile strength and elasticity [16] but also of the integrity preservation of skin, connective tissues, tendons and bones by mediating a fundamental inter- and intracellular signaling. The repetition of the Gly-X-Y sequence is indeed fundamental for collagen to properly perform its function and, to this, it remains almost unchanged during the course of evolution of the animal kingdom [18]. To this, mutations in the collagen *COL1A1* gene, have been associated to more than 400 human disease [19].

Because of the important role in cell signaling, the collagen triple helical molecule is characterized by the presence of a high number of integrin binding sites (i.e., the “GxOGER” sequence, where “G” is glycine, “O” is hydroxyproline, “E” is glutamate, “R” is arginine and “x” is a hydrophobic amino acid) fundamental for cells adhesion and interaction [20,21]. Therefore, non-structural functions of collagen are of great relevance for cell communication, proliferation, differentiation besides for healing processes [4,22,23].

The prevalence of collagen in human tissues and the important roles covered in the extracellular matrix (ECM), make it a natural choice for its employment as raw material [10]. Being the main component of the ECM, collagen is intrinsically biodegradable, biocompatible and bioactive [15,24,25,26,27]. Its abundance and ubiquity make it not perceived as exogenous constituent of the body [10]. As befits the primary structural protein in the body, collagen is naturally resistant to proteolysis but susceptible to attack of matrix metalloproteinase (MMPs) (i.e., MMP-1, MMP-2, MMP- 8, MMP-13 and MMP-14) [25,28,29]. The collagen fragments resulting from the action of collagenases, are further degraded by gelatinases and non-specific proteases. The presence of an accurate and complex degradation system for the endogenous collagen makes the exogenous collagen highly biodegradable [25]. Moreover, collagen and its degradation products could also promote the tissue’ structure and function restoration [30]. Lastly, collagen can be easily processed to fabricate several kinds of substrates like sponges, hydrogels, tubes, powders and films according to the final application [31].

All these attractive and advantageous features of type I collagen make it one of the most widely used biomaterial in health-related sectors, including medical care, pharmaceutics and cosmetics [32,33,34,35,36,37]. More specifically known is its employment as biomaterial for the manufacture of Tissue Engineering Medical Products (TEMPs) for tissues healing and regeneration. Moved by the great advantages in its use, various vertebrates have been extensively employed to isolate type I collagen. In spite of several attempts of extraction from different animal species, the best collagen sources are represented by mammals, such as bovine and swine, for the high sequence homology with human collagen [19]. However, the incidence of immune responses, the risk of zoonosis transmission and some religious concerns limited their use and favored horses use as a safer collagen source. Thus, the equine tissues appear as an attractive alternative, since they are almost free from zoonosis [38] and there are no documented immune reactions [39].

This review will specifically deal with scientific research carried on equine tendon collagen. Herein, the advantages and disadvantages in horse tendon use as extraction source are discussed along with its favorable biological properties and related applications. Accordingly, the last 15 years experimental studies and clinical trials on equine tendon collagen-based and approved devices for clinical use are argued. To the best of our knowledge, this is the first review that deal with only on horse tendon collagen-based products.

## 2. Why Equine Collagen

Type I collagen’s use as biomaterial for the manufacture of products related to the healthcare sector, the food industries and cosmetics is very high. The industrial production of collagen is based on its purification from animal tissues rather than from recombinant production systems [40]. The inability to reproduce the full-length collagen molecule with the native post-translational modifications (i.e., hydroxylation) decreased the interest in the use of both prokaryotic and eukaryotic hosts (i.e., yeast, bacteria, mammalian cells, insects or plants) for its synthesis [41]. As regards collagen extraction from animal tissues, several sources have been investigated [36], including mammals (bovine [42], porcine [43], ovine [44], equine [45,46], rat [47]), avian (chicken [48]) and fish (jellyfish, fish, sponges) [49], with the aim of finding the optimal one in terms of biocompatibility, safety and availability.

The highly available marine collagen, that has a lower threat of transmissible diseases and no religious concerns, is limited in its use in the healthcare sector because of its low denaturation temperature and enzymatic resistance [49]. On the other side, although the evolutionary closeness to vertebrates, poultry collagen molecule has an amino acid composition different from other mammals [50]. Moreover, the avian influenza transmission risk is not a negligible aspect [51].

**Figure 1:**
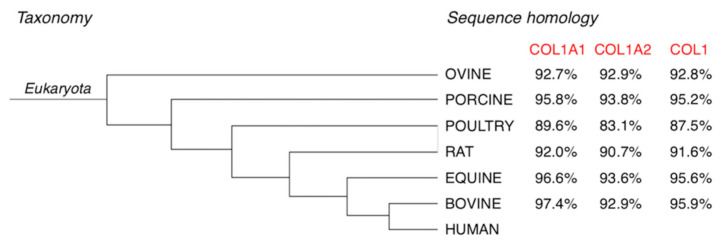
Taxonomy and sequence homology of selected mammalian collagen compared to human collagen. Identity percentages esteemed by collagen sequence alignment evaluation of α1 and α2 chains of equine, bovine, rodents, avian, swine and ovine in comparison with human collagen, by mean of UniProt (https://www.uniprot.org/align/) sequence alignment bioinformatic tool (last accessed on 3 April 2020).

Definitely, mammals represent the best source for the high sequence homology with human collagen (Figure 1) [19,52,53,54]. Moreover, the abundancy of waste materials (e.g., skin, tendons, bones, fatty tissues) from meat processing favored the exploitation of low-cost by-products for the purification a biomaterial with a high added value. The use of waste products for the extraction of a highly required product, such as collagen, not only makes discards valuable resource but also reduces their disposal costs and environmental impact. However, only in the last 50 years the use of heterologous collagen as medical product spread with the development of both accurate extraction processes that allowed removing allergenics and effective sterilization procedures [55].

Type I collagen could be isolated from several body districts. Among these, mammal skin and tendons are preferred due to the high protein yield [24,56]. As regards tendons, roughly 60%–85% of the dry weight is collagen [24,56] and type I collagen constitutes 90%–95% of the total collagen content [48,57,58]. To this, the lowest amount of protein contaminants is present in this district [59]. Otherwise, collagen content in mammalian dermal tissue is about 60%–70% and includes many other components such as blood vessels, lymph vessels, hair follicles and sweat glands [25,43], for which an accurate purification step is needed.

The extraction source not only influences the yield and purity of the final product but also its physical-chemical properties since the collagen structure and characteristics are deeply affected by the function it has in the belonging tissue [60]. The structure of tendon is such that the collagen fibers are aligned in the main load bearing direction and fiber diameter is larger than in skin [61,62]. The diameter and the orientation of fibers play an important role in tissue stability: a greater orientation of the fibers reflects a higher molecules compaction, resulting in a greater chemical-physical stability. Herein, collagen molecules were typically aligned and packed with a conserved stagger of 67 nm to form fibers with a medium diameter of 400 nm [46,59,63]. In skin instead, even if collagen is anisotropically distributed (along Langer’s lines) it is arranged in a loose network [60,64].

Besides, the collagen extracted from a tissue with a strict hierarchical organization, such as tendon, still retains a partial lateral packing arrangement despite the disruptive treatments of the extraction process [60,63,65]. The partial retention of the lateral arrangement of collagen molecules could also be ascribed to the well-known higher percentage of lysine and hydroxylysine in the α-helices of tendon collagen than in other tissues. These amino acids, fundamental for the intra- and intermolecular crosslinks, make tendon an extraction source of a type I collagen with superior properties over collagens derived from other tissues [56].

Thus, the highest type I collagen content and the lowest amount of protein contaminants in this body district [59], besides the appealing physical-chemical properties, make mammalian tendon as an attractive source for medical grade collagen. Moreover, mammalian tendons could be easily harvested from slaughterhouses without interfering with the meat harvesting process [56], while mammalian skins need to be appropriately separated from meat and hair.

Among mammals, bovine and swine are the most common extraction sources. The reason lies in the fact that these two species are the highest consumed mammalian meats per capita in the United States [31,66]. However, although bovine and porcine collagens cover most of the market size and tendon recover is easy, their use is limited because of immune response, zoonosis problems and religious constraints. Despite collagen is particularly poor immunogen [67] and the triple helical domains of bovine and porcine collagens are highly homologous to human collagen, immunologically relevant differences lay in the telopeptide regions [68]. Bovine collagen triggers immune reactions in about the 2%–4% of the World population [69]. However, this sensitivity has been considered generally acceptable for tissue engineered implants for human use [70]. Furthermore, the fact that up to 3% of the population manifests an inherent immunity [67,71], is enough to routinely perform allergy testing prior to material implantation [70]. To this, two consecutive negative skin tests at 6 and 2 weeks are required before any treatment [72]. Among issues, the zoonosis transferring risk (e.g., the foot and mouth disease (FMD) and the group of the bovine spongiform encephalopathies (BSE), among which the most dangerous for humans is the transmissible spongiform encephalopathy (TSE)) is the most serious. Porcine collagen causes less allergic response [36] but, just like the bovine source, the setback of zoonosis limited its use [73]. In addition, there are cultural or religious concerns associated with the use of porcine (Jewish, Islamic faith) and bovine (Sikh, Buddhism) collagen, which further restrict their applicative potential [34].

The ovine, a mammal of interest as dietary source of milk and meat, has no religious constrains but has the drawback of being susceptible to a special type of transmissible spongiform encephalopathy, namely scrapie. However, such a prion is known to not cause any diseases as the human-like variant Creutzfeldt–Jakob disease (vCJD), which is caused by BSE exposure to humans [74]. The only exploitable ovine source is the Australian one that is the sole disease and prion free in the world [75]. Holista Colltech, with a patent production process for ovine collagen, has the exclusivity to produce a zoonosis-free ovine collagen and does not have the adequate means (in terms of raw materials disposability) to sustain the World’s high demand of collagen-based products [75].

Rat tail tendon is one of the most commonly used source of type I collagen among researchers (in contrast to the industrial use), given the extensive amount of literature concerning isolation and characterization [40]. However, it is not used for the manufacture of medical products because of the unavailability of medical-grade type I collagen.

Conversely, horse-derived collagen is generally recognized as almost free from zoonosis transmission risks [38], with no reported immune reactions [39,57,76,77]. However, equine meat and thus equine collagen-based products are religiously not accepted by Jews and Muslims.

Although it is not well known, equids are also exposed to alphaviral equine encephalomyelitis (AEE), a mosquito-borne zoonotic infection that includes: (i) Eastern equine encephalomyelitis (EEE), (ii) Western equine encephalomyelitis (WEE) and (iii) Venezuelan equine encephalomyelitis (VEE) [78]. The AEE endemic life cycle involves different mosquito species (i.e., *Psorophora, Ochlerotatus, Coquillettidia, Ochlerotatus, Aedes and Culex*) and mammalian hosts (i.e., birds, rodents) to be spread to horses and other animals which are dead-end hosts [79]. Occasionally AEE can spill over to involve humans as dead-end hosts. In particular VEE often causes massive epizootics in horses and epidemics in human, whereas for EEE and WEE individual cases or limited outbreaks in both horses and humans were registered [78]. Some AEE cases have been reported in the past but in recent years, only few cases annually occurred and no epidemics have never been reported. However, should be noted that many cases may go unreported and undiagnosed since AEE infections usually are asymptomatic and encephalitis occurs in less than 4% of symptomatic cases [79]. Moreover, mortality rates of symptomatic cases are quite low (1%–7% for VEE and WEE, 50%–70% for EEE) [78,79]. Although the zoonosis transmission risk is not negligible, the occurrence of few encephalitis cases and the very low AEE-due mortality rate lead to the consideration of equine by-products as zoonosis-free extraction sources for medical-grade collagen.

Nevertheless, compared to collagen from other mammals, equine collagen is characterized by the highest homology sequence with human collagen, after bovine (Supplementary Figure S1). The high percentage of sequence alignment is due to the taxonomical closeness of equines and bovines to humans (Figure 1). The low evolutionary gap and the high conservation of type I collagen amino acid composition among vertebrates make that homology up to 95%. Thus, equine collagen, that compared to bovine is equally similar to human collagen from a compositional point of view, seems to be a valid alternative to bovine collagen.

Moreover, as before mentioned, collagen extracted from a tissue with a strict hierarchical organization, such as tendon, is characterized by a higher percentage of lysine and hydroxylysine than other tissues. Interestingly, collagen from equine tendon revealed to have the highest lysine and hydroxylysine level compared to those extracted from other mammal tendons (Table 1). The peculiar amino acid composition of equine tendon collagen and the related stronger fibers packing is the reason why devices manufactured with native horse tendon collagen are intrinsically more resistant to degradation and mechanical stress [63]. As reported by Angele et al., equine tendon collagen compared to bovine tendon collagen was found to have a higher thermal stability and a tendency to rupture under higher mechanical resistance [45].

The partial preservation of fibers packing [63,81], is a key aspect because it influences not only bioengineering parameters but also the cell-biomaterial interaction since the nanometric fibril organization is recognized by cells as guide for cell growth and migration during the remodeling phase of the healing process [26,27,60,82]. By the way, it should be noted how despite collagen extraction protocols are set up in order to preserve its native structure as possible, the application of mechanical, chemical and enzymatical treatments brings to a partial de-structuration of the strict hierarchical organization of collagen fibrils. In particular, the enzymatic treatment cuts collagen molecules at the N− and C− termini, modifying their native state and making them more susceptible to enzymatic digestion and thermal denaturation. Thus, while type I collagen fibrils are regularly packed in tendon, the isolated ones are characterized by smaller diameter and length and thus by lower mechanical properties. Moreover, it should not be neglected that collagen extraction sources are animal tissues and various factors, such as animals age, sex and inter-species variability, make collagen chemical and physical properties not punctual but within a range of values.

Thus, the structural organization of the native tissue is not completely preserved in the extracted product. About this topic, some attempts were made in order to in vitro reorganize collagen fibrils (i.e., fibrillogenesis) in fibers that could resemble the natural tendon ones. Although a partial alignment could be obtained, to date it is not possible to completely reassemble the extracted tendon collagen fibrils in vitro, in the ordered hierarchical organization naturally present in tendon.

Nevertheless, the strict hierarchical organization of equine tendon, compared to other horse tissues and tissues form other mammals, allows to better retain collagen native structure after the extraction process and the following processing [60]. However, should be noted how, to the best of our knowledge, few data were reported about the comparison of equine tendon collagen properties with collagen from tendon of other animals with the same extraction method and substrate synthesis protocol applied. Moreover, the patent-due confidentiality of the collagen isolation protocols hinders such comparison since processing variations strongly influence the final products properties. Although the lack of exhaustive, numerous and public supporting data, the advantages offered by the use of equine collagen as biomaterial are visible from its employment by several well-known Companies (as argued in Section 4). Definitely, the horse tendon with its structural interesting features and the freedom from the afore-mentioned source-related issues would be considered as a valid and alternative extraction site of medical grade type I collagen.

## 3. Equine By-Products Accessibility

**Figure 2:**
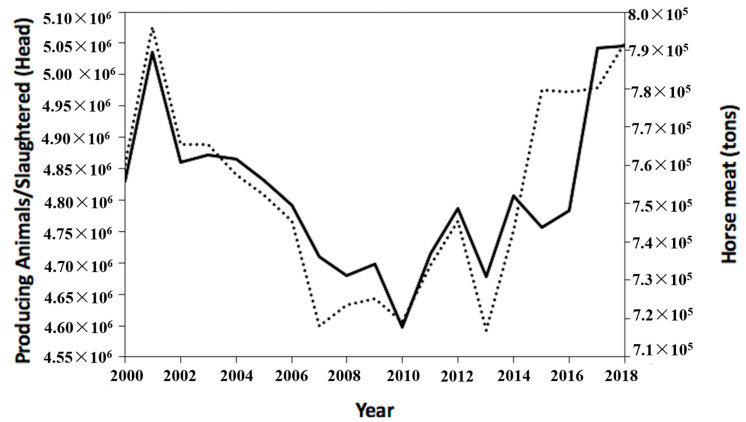
Global consumption of equine meat per year. Worldwide slaughtered horses (line) for the production of horse meat (dot) for human consumption per year. Data obtained from Food and Agriculture Organization Corporate Statistical Database (FAOSTAT) (http://www.fao.org/faostat/en/#data) on-line information system (last accessed on 10 July 2020).

As previously argued, collagen is usually extracted from meat processing waste by-product. By comparison to other meat producing species such as porcine, poultry, bovines or ovine, horse meat production represents only 0.25% of the total worldwide meat production [83,84]. The low consumption of horse meat is due to cultural, economic and social reasons that strongly impact on eating habits. Traditionally, horses have been employed over the years as farm workers, companions for recreations or sporting reasons. This historical association and the related positive emotions generated, such as affection, closeness or tenderness, led to the adoption of horses as a pet in many cultures and thus to the rejection of equines as a source of meat for human consumption [83,84]. Additional constraints are imposed by the Islamic and Jewish religion for which horse meat is forbidden (*haram*). At the same time, equids meat is also the center of the controversial issues of the economically-motivated improper adulteration of food. The horsemeat scandal in 2013 negatively affected the image of equine meat and led to a boycott of horsemeat-based products by some consumers, resulting in a reduction in sales of processed meat [84,85]. Apart from cultural, social and religious limitations, horse meat forms a significant part of the culinary traditions of many countries, particularly in Europe, South America and Asia for which large amounts of horse meat, such as an average of 750,000 tons of meat for year, are consumed. The recent reconsideration of equines as an industrial meat source is correlated with the BSE spread. Thus, although meat production from horse is still lower than that registered for other species, FAO data reported a crescent horse slaughtering (about 5,045,488 of slaughtered horses in 2018) with a proportional increase in horse meat consumption that reached a value of about 800,000 tons in 2018 (Figure 2).

Horse meat consumption is not only affected by cultural, social and religious factors but also by strong regulations. Horse meat and its by-products can be considered as legitimate food ingredients or products for human consumption only if appropriate actions are taken such as veterinary inspections and slaughter in approved and certified slaughterhouses [84]. Should be noted that not all equines could be slaughtered for the production of meat for human consumption. Horses that are not bred for food (i.e., equids for sport competition) that are regularly administered with drugs or other chemicals are forbidden. Moreover, horses that have been euthanized by mean of lethal intravenous injections of drugs (i.e., sodium pentobarbital) could not be used for meat production. The potential danger of eating meat contaminated with such chemicals is still completely unknown. To this, the EU, the FDA and the USDA prohibit the presence of many everyday equine drugs such as antibiotics, anesthetics, anti-inflammatories, de-wormers in meat for human consumption.

As well as horse meat, also by-products follow the same regulation and restrictions. Horses tendons selected for the extraction of fibrillar type I collagen meant to be used as raw material for the development of biomedical products or food supplements for human use, do not derive from animals that have been euthanized by mean of a lethal injection or sport horses to whom drugs or other substances were administered. Each by-product batch is therefore subjected to strict inspections and it is supplied with official conformity certificates. The presence of only certified drug-free meat and by-products for human consumption allow to have a sufficient availability of waste material eligible for collagen extraction. Moreover, the recent increment in horse meat consumption directly increases the availability of by-products.

Although horse meat historically came from old animals used for farm-working, nowadays it is generally supplied by young animals bred for this purpose [83]. The request of a certain the degree of tenderness is indeed a significant age-related aspect of meat quality. This trend is in accordance with the needing of young horses for the extraction of collagen from tendon. In particular, equids of 18–48 month were preferred. The maturation of equine tendon collagen fibrils during the growth strongly impacts on collagen features such as solubility and reactivity and thus significantly influences the final product properties. The increase of intramolecular and intermolecular cross bonds with age make collagen fibrils even more insoluble [86] and consequently less reactive due to the reduction of available reactive groups. The strong fiber packing makes collagen fibrils less susceptible to the enzymatic proteolytic attack and to acid or alkaline treatments, fundamental extraction process steps. Thus, the age-related maturation of collagen makes extraction from tendons of old equids more difficult and with a lower yield than that one achievable from young individuals. To this, tendons of young animals are preferred and usually selected for this purpose.

## 4. Currently Approved Equine Collagen-Based Devices

The use of xenogeneic collagen as a modern biomaterial began in 1881 when Joseph Lister and his former student William Macewen independently reported on the *British Medical Journal* the advantages of a biodegradable suture termed “catgut” derived from the small intestine of a sheep [87]. From that moment on, the idea of exploiting xenogeneic material for human surgical practices spread to the scientific community. The high conserved compositional similarity among mammals is a strong point that could be exploited to reach a better natural-like tissue healing [53]. Citations dating to the 1940s and 1950s relates to experimental attempts of purified collagen implantation in animals [88]. Only 30 years later, the first medical use of collagen in humans was reported by Knapp with an injectable collagen gel formulation for soft tissues augmentation [89]. In 1980, one of the first mammal collagen formulations (i.e., Zyderm^®^ by McGhan Medical Corporation, Fremont, CA, USA) started to be commercialized [90]. Over the ensuing years, countless collagen-based formulations were manufactured with the aim to restore or repair soft and hard tissues physiological function [91,92].

The history of implantable collagen-based products let us know about the high interest that turns around it. Therefore, it has always been a target not only to isolate collagen from animal tissues but also to obtain a safe xenogeneic product, which meets regulatory requirements and which can be implanted without triggering unwanted reactions. For instance, medical devices to be commercialized should meet the essential requirements defined in the Annex I of the Council Directive 93/42/EEC (which is going to be replaced by the Medical Device Regulation (MDR) 2017/745 in May 2020) [93]. The manufacturing process of a device, including all aspects going from the raw materials to the delivery of the final product, should be fully validated to ensure reproducibility and safety for human use.

Among the various aspects, the approved for human use products must above all be free from allergens or toxic compounds that could trigger immune response. Even if collagen is typically low immunogenic, other ECM proteins (i.e., DNA, RNA, cells remnants, α-gal epitope and MHC-1) are able to evoke immune response, adverse reactions and rejections [70,94]. Since immunogenicity is the primary cause of immunotoxicity, the immunogenicity evaluation is a critical but essential aspect for collagen products. A not-negligible aspect is the material contamination by bacterial endotoxins (i.e., lipopolysaccharides), that are components of the external cell membrane of Gram-negative bacteria able to stimulate the inflammatory response at very low doses (0.5 EU/mL) [95,96].

Another reason why collagen products could evoke adverse effects is the crosslinking, in particular chemical crosslinking. Physical crosslinking as the dehydrothermal treatment (DHT) instead is safer and biocompatible [97,98]. During resorption, chemical crosslinking likely affects MMPs bioactivity against native collagen, producing an imbalance in ECM turnover [70]. The delayed resorption and the substrate inertness to degradation prolongs implant presence in the tissue, exacerbating host responses to the implant. Additionally, not-natural collagen degradation fragments, bearing remnants of added synthetic chemical crosslinkers, are recognized as antigens and amplify the foreign body response [99,100]. That is why almost all commercial products are not chemically crosslinked.

Devices sterilization is the last key process to accurately set prior to the products packaging. Collagen is a temperature sensitive biomaterial that could not be autoclaved. For this, alternative sterilization processes have been investigated but until today the ideal technique has not been identified. Any known sterilization technique induces molecular alteration to collagen triple helical structure with a consequent decrease of properties such as the mechanical and the enzymatical resistance [101]. However, some methods are more permissive than others. Ethylene oxide sterilization and β-ray irradiation induce less damage than γ-ray but their applicability depends on the type of collagen-based device to be produced [91,101]. The preservation of the native collagen structure as much as possible among the whole manufacturing process is preferred since it accelerates the regeneration stage, shorts the wound healing time, reduces the extent of bacterial contamination, alleviates the pain syndrome and reduces the recurrence rate [102].

To date, numerous preparations based on equine tendon collagen received the approval of the US Food and Drug Administration (FDA) for human use and are commercially and clinically available. From 1990 onwards, the date at which the first device was registered based on horse tendon collagen for wound healing (Condress^®^ now called Biopad^®^ by Euroresearch), companies like Baxter, Bioteck, Euroresearch, Finceramica, Fidia Farmaceutici, Innocoll Pharmaceuticals, MLM Biologics, Nycomed, Opocrin, Resorba, Savecoll, Takeda, Vebas manufactured and commercialized devices based on equine tendon collagen with several patented techniques (Table 2).

**Figure 3:**
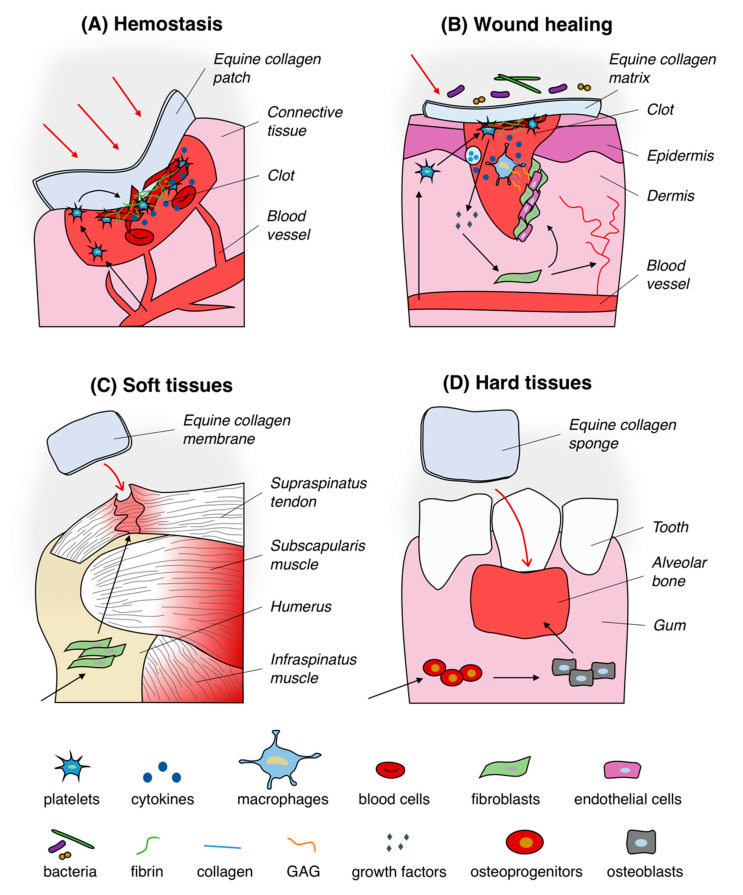
Typical uses of equine collagen-based products in biomedical applications. Equine collagen-based products are usually used as hemostatic agent (**A**), wound dressing (**B**), matrix for soft (**C**) and hard (**D**) tissues regeneration. Black arrows in section (**A**) and (**B)** indicate the trigger of the hemostasis process and the healing of wounds pathway by mean of equine collagen substrates, respectively. Black arrows in section (**C**) and (**D**) represent the equine collagen matrix-mediated enhancement of soft and hard tissues regeneration process, respectively.

Thanks to its intrinsic biocompatibility [26,27] and regenerative properties, equine tendon collagen-based devices have been manufactured and applied in relation to a variety of medical applications (Figure 3) such as in reconstructive surgery to speed up wounds closure, to regenerate burned skin and soft tissues as well as to guide bone and cartilage repair. Between the listed ones, the use as hemostat is one of the most important application.

### 4.1. Hemostatic System

Effective hemostatic systems for traumatic or surgical lesions of soft tissues such as viscera, liver [143,146,147,171], heart [106,145,151,155], lymphatic system [150], kidney [144,148], post-cesarean uterus [149], blood vessels [139,142], have always been of great importance. During surgical interventions on parenchymatous organs, hemorrhages are hard to control. Apart from different surgical measures to stop such bleedings (compression, ligation, suturing, clipping argon beam coagulation, electrocautery), a very common strategy consists in the use of topical agents with high hemostasis power (collagen-based sealants, cellulose fleece, cotton gauze, synthetic glues, fibrin sealants) [145]. Among these, promising results were given by collagen-based pads that demonstrated to be able to promote coagulation within few minutes after the application (3–5 min) [143,171] by naturally favoring platelets recall by chemotaxis besides their adhesion and aggregation. The contact of platelets with collagen start a reaction cascade which leads to platelets aggregation to form a clot that effectively reduces bleeding [82]. It is worth noting that the cascade of events can be induced by native collagen rather than its denatured form [82,171], reasons why all hemostatic commercial products (TissueFleece^®^, Biocollagen^®^, Tachotop^®^, Antema^®^, CollGARA^®^, Kollagen^®^, Parasorb^®^) take care in maintaining the nativeness and thus the natural binding sites of collagen during the manufacturing process.

The intrinsic adhesiveness of collagen demonstrates its efficacy in the treatment of wounds not only by promoting good hemostasis but also by favoring repairing processes [171]. This “secondary” property of collagen is important to decrease the probability to have surgical site infections. Post-operative complications often require additional surgery besides a significantly longer hospitalization, increasing medical care costs and patients’ morbidity and mortality rates [155]. In this scenery, an equine tendon collagen sponge enriched with the antibiotic gentamicin (i.e., Genta-coll^®^, Gentafleece^®^, Septocoll^®^, Collatamp^®^) significantly reduces post-surgical infections and related morbidities while performing its main hemostatic function [106,118,129,130,155].

The efficacy of some horse tendon collagen-based products for hemostasis was upgraded by the addition of coagulation factors such as thrombin and fibrinogen (TachoSil^®^, TachoComb^®^) in order to be superior to the standard hemostatic suturing, argon beamer coagulation and conventional hemostatic materials [139,140,142,144,145,146,147,150,151]. Upon the contact with body fluid the clotting factors of fibrinogen and thrombin dissolve and form a fibrin network, which glues the equine tendon collagen sponge to the wound surface [142,150]. The application of an equine tendon collagen sponge enriched with fibrin/thrombin decreases both post-operative complications, blood transfusion and, consequently, hospital stay and addressed medical costs [146].

Apart from hemostasis, equine tendon collagen-based substrates (TissuFoil^®^, CollGARA^®^) have been successfully evaluated also as protective barrier between the polypropylene mesh implant and abdominal organs with the aim to separate the adjoining tissues and organs in the areas of the abdominal cavity [107,169].

### 4.2. Healing of Wounds

Initially developed to act as a barrier against a harmful external environment [127], equine tendon collagen formulations (e.g., sponge, foil, membrane) revealed to be suitable as tissues temporary substitutes. Collagen, with its ability to absorb and retain a large amount of fluids, provides for a moist environment that promotes wound healing [77,172]. A number of biochemical, histological and immuno-histochemical investigations revealed how several pro-regenerative activities are mediated by collagen (proteases inhibition, vascularization promotion, fibroblast growth) [173,174].

Native collagen interaction with platelets results in the formation of a clot that provides a matrix for the influx of inflammatory cells and in the secretion of growth factors like the platelet-derived growth factor (PDGF), the transforming growth factor-beta (TGF-β) and the endothelial growth factor (EGF) [40]. While the PDGF in conjunction with proinflammatory cytokines contributes to attract neutrophils for bacterial removal, the TGF-β contributes to convert monocytes into macrophages, which initiate the development of granulation tissue and release various proinflammatory cytokines (e.g., interleukins 1 and 6 (IL-1, IL-6)) [40]. TGF-β and PDGF play a key role in the conversion of fibroblasts into myofibroblasts, which generate contraction forces that facilitate the wound closure [40]. The formation of the granulation tissue is of fundamental importance for the synthesis, deposition and organization of a new collagen-rich ECM. An in vivo study on epithelial cells derived from dermal microvessels demonstrated how type I collagen activates angiogenesis, a fundamental process to reach a good level of tissue regeneration [102]. The absence of blood vessels is indeed the first cause of regeneration processes failure since no cytokines, growth factor, cells and nutrients could reach the injured site.

Another important role of native collagen, especially when dealing with chronic wounds, is its ability to inhibit proteases and cytokines (neutrophil elastase, MMP-2, IL-6, IL-8, IL-1, superoxide-anion, peroxynitrate) by mean of its high binding capacity for them [102,175]. Chronic wounds contain elevated levels of pro-inflammatory cytokines like IL-1β and TNF-α, elevated release of reactive oxygen species (ROS) by neutrophils and increased expression of proteases that lead to severe tissue damage and impairs wound-healing [175]. Hence, the reduction of the level of MMP and IL in wounds bed is fundamental to avoid the breakdown of growth factors and other agents that stimulate native fibroblasts to produce the granulation tissue, a key step in wound healing [136,172].

Usually, collagen dressings are manufactured essentially from the tendon of horses (Biopad^®^, Bionect^®^, Collexa^®^, Revamil^®^, Bio-ConneKt^®^) and bovine, as well as from the skin of bovine and pigs [174]. However, the choice of the collagen origin influences the wound dressing performance. Collagen of various origin exhibits a different binding capacity for IL-1β and TNF-α and elastase. As regards TNF-α, bovine collagen exhibited the best binding capacity in vitro, followed by equine and porcine collagen that showed a comparable capacity [172]. In the case of IL-1β, the binding affinity of bovine collagen is followed by the one of equine and lastly of porcine collagen [172]. Elastase levels instead are likewise reduced by bovine and equine collagen [175].

Although bovine collagen seems to have the best in vitro response, equine collagen-based wound dressing formulations exhibit the best structural compromise when compared to analogues devices made of bovine or porcine collagen. A comparative study of Karr et al., demonstrated equine tendon collagen pad to be the only product that retains the same overall structure during exposure to collagenases and at the same time to allow the collagen interlaced matrix to be clearly frayed off [127]. The maintenance of the structural integrity for a longer period may be a key condition for a better healing process by mean of a longer native collagen-wound interaction. In the comparison between commercial products, the fact that collagen-based products are not all the same could not be overlooked. Known is how differences in composition and degree of preservation of the natural collagen matrix strongly influence the final product clinical effectiveness [102].

More recently, an equine tendon collagen injectable gel (Salvecoll^®^) was proposed for the post-surgical treatment of fistulas with the aim to eradicate sepsis, mechanically fill the defect and promote healing by providing a transition matrix [100]. The invasion of the collagen gel by fibroblasts from the surrounding tissues stimulates the immune system and thus the release of growth factors fundamental for tissue healing [100].

Despite the differences in attenuating the chronic inflammatory response and in favoring healing, all mammal-derived collagens own such intrinsic properties that are not achievable by any other material [174]. Traditional dressings (paraffine gauze, cotton pad, rayon/cellulose sandwich) have very high absorption capacity but they cause rapid dehydration. Their daily removal from the wound surface can cause bleeding and damage of the newly formed epithelium [90,174]. In addition, one of the most significant problem encountered in traditional dressing is the foreign body reaction in the wound caused by cotton fibers [90]. Collagen-based dressings instead do not need to be removed and can be left on the wound for up to seven days [174]. Exceptional is the healing time in the case of diabetic foot and heel ulcers that with an equine tendon collagen sponge occurs in approximately 31 days [176]. Even if collagen wound dressings are much more expensive than traditional gauze and pad, a faster healing process is obtained as well as less frequent dressing change [174]. The initial higher expenditure is therefore mitigated by the faster and better patients’ recovery.

Advanced and still experimental approaches for the treatment of wounds were offered by tissue engineering overture. Several research groups reported studies on equine tendon collagen products meant to be used as carrier for cells [109,117,161], drugs [162] or genes [138], with the aim to bettering injured tissues’ regeneration and physiological functions restoration. Few cell populations such as adipose stem cells [117], adipose tissue stromal cells [161] and fetal skin cells [109], have been investigated. As regards burns, the combined use of a biological bandage and autologous cells helps in the overall skin repair and soft tissue reconstruction. For this, adipose stem cells isolated from patient’s tissues were integrated within an equine tendon collagen scaffold to provide a better cell delivery system to the burned site. Studies of Krahenbuhl et al. demonstrated how adipose stem cells already integrate into the matrix at 24 h and, after 48 h, strongly adhere and migrate within the scaffold [117]. A similar approach was followed by Hohlfeld et al., that treated pediatric burns with equine tendon collagen sheets seeded with fetal skin cells [109]. The powerful proliferation and migration ability of fetal skin cells led to a high-quality skin recovery just after two-week treatment without the addition of fixative (i.e., glue, staples, stitches, silicones) and anesthesia [109]. A better wound closure was obtained compared to the traditional autograft method that not only needs more healing time (three weeks) but often involves the formation of an hypertrophic granulation tissue [109]. With the aim to improve equine tendon collagen-based scaffolding properties in wound healing by avoiding excessive scar formation, an attempt was done by embedding drugs. Garrier et al. pre-clinically evaluated the impact of a photodynamic liposomal formulation (Foslip^®^ by biolitec research GmbH, Jena, Germany)) embedded in an equine tendon collagen scaffold on healing processes with promising results [162].

### 4.3. Soft Tissues Regeneration

Less and still experimental are healing attempts of human soft tissues such as tendon [58,177,178,179], peripheral nerve [105,115] and adipose tissue [161]. Notwithstanding, clinically approved are equine tendon collagen devices for dura mater [111,112,113,114] and cartilage [123,124,131,132,133,134,141,180,181,182,183,184] repair.

Tendon disorders are responsible for marked disability that strongly impacts on patient quality of life. Despite a high number of attempts, the regeneration of this tissue by mean of a temporal substitute is still one of the biggest challenges in tissue engineering. Collagen is the main load-bearing component in the natural ECM by conferring mechanical strength required for load-bearing [58]. The loss of mechanical function in repaired tendon is due to formation of a distorted ECM with misaligned collagen fibers [123]. The use of equine tendon collagen scaffold in which collagen maintains an aligned structure could offer a chance. After assessed scaffolds suitability for human tendon regeneration [153], electrostatically oriented multilamellar equine tendon collagen membranes in vitro confirmed how fiber orientation provides an instructive pattern for cell growth and drives cell alignment mimicking that seen in normal tendon [177,178]. However, leading towards a partial tendon healing, this kind of substrate was suggested for human tendon augmentation rather than a tendon temporal substitute [177]. One of the problems encountered in developing an equine tendon collagen-based device for tendon regeneration is the low mechanical strength compared to that of the native. An attempt to increase the low order magnitude of collagen in terms of mechanical properties was done in a pilot in vivo study performed with a core-shell scaffold made of equine collagen, 1,4-butanediol diglycidyl ether (BDDGE) and elastin [179], with promising results.

The restoration of peripheral nerves function after traumatic injuries is another challenge for tissue engineering. Equine tendon collagen sponge revealed to be able to in vivo improve motor function and proprioceptive recovery [115]. However, regenerating axons did not penetrate the scaffolds in their full length and thus did not contribute to the significant improvement of functional recovery. An attempt to improve nerve regeneration after end-to-end reconstruction was in vivo performed by enwrapping an equine tendon collagen scaffold seeded with N1E-115 neural cells around the lesion site, without successful results [105].

Moreover, equine tendon collagen scaffold could serve as carrier of adipose tissue. Although adipose tissue is a less frequent target, it is highly required in plastic and reconstructive surgery, especially facial [161]. Besides its essential metabolic functions, adipose tissue provides the shape and volume of the outer body contour and preserves the mobility of tissue layers [161]. The implantation of equine tendon collagen microcarriers charged with adipose tissue stromal cells could be a resolutive strategy in the case of adipose tissue lack in congenital disorders, in aesthetic surgery and after tumor ablation [161]. However, with the aim of biorestructuring the dermis for aesthetic purposes, a recently available cell-free injectable equine tendon collagen formulation (Nithya^®^) showed clinical efficacy [39].

The dura mater is a thick membrane that surrounds the brain and spinal cord whose function is to mechanically protect tissues present below. After cranial, spinal and trans sphenoidal neurosurgical procedures, the correct reconstruction of the dura mater is needed. The application of an equine tendon collagen foil (TissuDura^®^) without suturing revealed to be the best choice for dura mater reconstruction since no local toxicity or complications (i.e., cerebrospinal fluid leaks, adherences or inflammation) were observed [113,114]. Moreover, the full degradation of the equine tendon collagen matrix and its replacement with native collagen neodura with neoangiogenesis formation was observed after 12 months [112,114]. Compared to other dural substitutes, one of the main benefit of equine tendon collagen foils is the transparency that allows to inspect the operative cavity after implantation and to easily permit an eventual second surgery [113]. Moreover, equine tendon collagen foils resulted to be easily adaptable, impermeable to external agents and hemostatic [111,112], ideal features for implantable devices.

Cartilage is often exposed to traumatic, inflammatory or degenerative injuries who led to disability and pain. Current strategies for the regeneration of cartilage usually involve the use of hyaluronic acid-based formulations for its viscosupplementation properties and collagen (MeRG^®^, MaioRegen^®^) for its pro-regenerative properties [182,185]. The needing of a regenerative support is due to the fact that the intrinsic healing potential of the damaged cartilage is limited because of the absence of blood vessels and innervation [180]. The missing of vascularization stimulation properties is the reason why most of the products on the market fail to provide long-term results [183]. Nevertheless, equine tendon collagen scaffolds in vitro revealed their potential in cartilage regeneration by promoting and supporting chondrocytes growth and proliferation [141,180,181,183,184]. Various cell populations seeded and cultured on pure type I collagen, multiplicate and express chondrocyte cells phenotype (i.e., expression of vimentin, Sox9, Matrilin-1, S100, CD99, aggrecan, collagen type II), accompanied by the production of ECM (i.e., immunohistochemical analysis on collagen type I, type II and proteoglycans) [180,181,183]. More than chondral, the osteochondral lesions are even more problematic since they involve two different tissues. To this, a novel multilayer scaffold made of equine tendon collagen and hydroxyapatite (MaioRegen^®^) successfully induced the healing of the chondral and the deep osteochondral defects in animal models, probably by inducing the selective differentiation of resident progenitor cells [131,132]. Promising results in vivo were clinically confirmed by equine tendon collagen-based scaffold implanted in knee defects [123,124,133,134,182], even if many doubts still exist regarding the ability to support both hyaline cartilage formation and subchondral bone ingrowth [134].

### 4.4. Hard Tissues Regeneration

The ECM of mineralized tissues such as bones and teeth consists of organic (35%) and inorganic (65%) phases, where the predominant organic protein, type I collagen, drives the heterogeneous nucleation of hydroxyapatite nanocrystals onto its fibrils, by activation of specific control mechanisms [186]. Hard tissues’ healing process is a complex biochemical procedure that involves the migration of osteoblasts, the formation of new blood vessels, the synthesis of collagen and new ECM and the cooperation of a variety of cytokines. In this scenery, blood vessels provide a conduit for the recruitment of cells involved bone deposition and are therefore a crucial condition for tissues regeneration [160]. Osteogenesis and angiogenesis are clearly linked in a strong codependent relation.

As regards dental care, equine tendon collagen products effectively reduce bone resorption after teeth removal and induce bone regeneration with a bone structure similar to natural [167]. To this, a high number of equine tendon collagen products (t-Barrier^®^, Biocollagen^®^, Xenomatrix^®^, Bio-gen^®^, Bioart^®^, Kollagen^®^, Parasorb^®^) are actually clinically used for the treatment of periodontitis [121,126], alveolar ridge preservation after tooth extraction [77,168], edentulism [125], maxillary sinus lift [119], intraoral donor sites regeneration [164], root perforation [163] and radicular cyst [120].

In periodontitis, collagen sheets are successfully applied on the bone root to increase the amount of periodontal connective tissue attachment, with new cementum and new bone formation, in order to decrease the pocket depth and restore the gingival recession [121,126]. After tooth extraction, bone resorption is avoided by improving alveolar bone regeneration by mean of an equine tendon collagen sponge that, by the formation on a stable blood clot, gives a natural support for osteoid differentiation and the subsequent calcification [119,168], besides guides epithelial and connective cells important for the post-extraction soft-tissues healing [125]. The enhancement of the bone strength in sockets or bone gaps by collagen may be due to its intrinsic angioconductivity and osteoconductivity [77,119]. The positive effect on alveolar bone healing derived from the application of an equine tendon collagen devices suggests its routinely use in the socket after teeth extraction [77].

Recent studies investigated the possibility of enhancing bone regeneration properties of equine tendon collagen with the addition osteoinductive molecules (such as growth factors or genes) [160,165] or by blending with other osteoconductive biomaterials [128]. The combination of an osteoconductive scaffold with osteoinductive protein such as the vascular endothelial grow factor (VEGF) better stimulates and supports bone healing and regenerating processes [158,160]. Some attempts were made by combining the bioactive properties of collagen with the osteoconductive properties of the poli-(L-lactic) acid [128] and of the hydroxyapatite [128,186,187], with promising results. Not negligible is the tissue engineering-based approach for which adipose tissue-derived stromal cells were seeded on equine tendon collagen scaffold in order to induce osteogenic differentiation [159].

Even if the replication of the natural mineralized tissues is still a challenge, these reported studies demonstrated the feasibility in developing mechanically stable scaffold with the ability to mimic the physical-chemical features of the natural bone.

## 5. Equine Collagen-Based Device Market

In the last 15 years, with the development of regenerative medicine and tissue engineering, collagen has been defined one of the best scaffolding materials, being biocompatible, biodegradable, bioactive besides easily manufactured. The remarkable advantages offered by this extraordinary and archaic natural protein means that the demand for collagen and collagen-based products never fades, rather it tends to increase with the increasing need of new effective and advanced therapies [188].

The native collagen market size was globally esteemed to be around USD 160.5 Million in 2018 [189]. Among the several application sectors in which collagen market is divided, the healthcare is the largest application area, followed by food and cosmetic. Healthcare dominates the collagen market with about 50% share of the entire market volume in 2025 [189,190]. Herein, in 2014 it has been esteemed a global addressable market of c. $16 bn at the end-market price, by counting c. $14 bn for bone graft and advanced wound healing, c. $1.2 for regenerative medicine scaffold and c. $0.2 bn for in vitro diagnostic [190]. The esteemed market should increase over years since the request of collagen for medical devices and drug delivery systems is expanding together with the trend towards minimally invasive technologies and its effectiveness in wound healing. Indeed, as regards tissue engineering products, the esteemed global market of $1.5 bn in 2014 [190], nearly doubled to c. $2.4 bn in 2017 with an expected compound annual growth rate (CAGR) from 2017 to 2025 of 10.4% [191,192].

To the best of our knowledge, even if no specific information on equine tendon collagen market are available, the interest in horse tendon collagen and derivates is clearly visible not only from the number of scientific researches but also from the number of patented manufacturing processes on equine tendon collagen and equine tendon collagen-based devices for biomedical and cosmetic application [193]. The long-time search for better strategies, the advanced manufacturing techniques used, the in-depth investigations on the properties of the products, the functionality checks (in vitro and preclinical testing), the safety assurance and the management costs gave to the healthcare products a high final cost. In general, no medical grade collagen-based products have been found worth less than $10,000 USD/kg [75]. However, even if the high final cost of all collagen-based products could be limiting, the ratio between costs and benefits should be considered. As afore mentioned, tendon collagen-based devices are able to promote natural healing processes faster and better than other biomaterials on the market [174]. Faster healing is associated with a lower risk of developing post-treatment or post-surgical complications, for which further treatments or second surgeries are needed. The decrease of healing time and complications rate reduces the needing of additional treatments, drug therapies and surgical procedures and consequently positively impacts on the patient’s psycho-physical state. Moreover, not negligible is the benefit in relation to other cost drivers such as hospital inpatient stays and personnel costs.

## 6. Concluding Remarks

Collagen has always been employed for several reasons. In ancient times, its use is recorded as the main ingredient of glues formed from the boiling of animals’ skin, tendons and ligaments. Only in the 19th century the world “collagen,” from the Greek κόλλα (kólla, “glue”) and -γενής (-genés, “-forming”), was coined to designate that constituent of the connective tissues that yields gelatin after boiling [194]. The use of collagen as a modern biomaterial began in 1881 [87] and pre-clinical trials with collagen-based products started around the 1940s [88]. However, studies on purified collagen use began in the late 1950s, after the development of collagen dissolution (i.e., solubilization) methods [92]. From 1959, when Pappas and Hyatt used film of reconstructed solubilized collagen for wounds healing in mice for the first time [195], preclinical and clinical studies on collagen-based devices for healthcare never stopped and continue nowadays.

The use of heterologous collagen as medical product really spread in the last 50 years thanks to the development of both accurate extraction processes and effective sterilization procedures [55]. Advances in purification processes allowed to realize collagen preparations with minimum immunogenicity both with high purity levels. Dilute acetic acid, pepsin digestion, filtration to remove impurities/insoluble matter, salt precipitation and dialysis are crucial steps in the production of high purity and low immunogenicity collagen [10].

Accordingly, mainly because of the BSE transmission risk from bovine collagen, the need of a xenogeneic product as safe as possible pushed towards the identification among mammals of horses as the best issue-free extraction source. The absence of immune reaction is another not negligible advantage of equine collagen.

Besides, regarding the extraction site, tendon represents the body district with the highest content of type I collagen and the lowest amount of contaminants [24,48,57,58,59]. The choice of equine tendon as collagen extraction source has remarkable advantages not only in terms of safety but also in terms of chemical-physical and biological properties of the purified raw material. Despite the disruptive treatments of the extraction process, horse tendon collagen retains a partial lateral packing [46,59,60,63,65], thanks to the higher content of lysine and hydroxylysine compared to other mammal tendon collagens. This feature makes collagen and collagen-based devices from horse tendon intrinsically more resistant to degradation and mechanical stress [45,63]. Moreover, the preservation of the native structure and packing influences not only bioengineering parameters but also the material bioactivity since the integrity of native cell-binding sites is fundamental of cell recognition [26,27,60,82]. Indeed, differences in the efficacy of native collagen-based devices not only depend from manufacturing processes but also from the percentage of preserved protein structure [62].

Equine tendon collagen devices have anti-inflammatory, analgesic and hemostatic effects, besides the ability to hinder the entry of outer microorganisms, to protect the wound, to restrict edema and fluid loss, to stimulate angiogenesis, osteogenesis and the migration of fibroblasts. All these features belonging to no other material make collagen one of the best biomaterials for the manufacturing devices for biomedical applications. For this reason, many native horse tendon collagen-based devices are currently successfully used in reconstructive surgery for the treatment of burns, trauma, infectious and surgical wounds. The appearance on the market of equine tendon collagen and equine tendon collagen devices suggests how this biomaterial is even more gaining the attention of the scientific community in all healthcare-related purviews such as medical, pharmaceutical and cosmetic sectors.

## Figures and Tables

**Table 1:** Comparison of the amino acid composition of type I collagen extracted from tendon of the most commonly used mammalian species in comparison with human collagen.

Amino Acids	Equine [45]	Bovine [45]	Ovine [74]	Rat [80]	Human [52]
Alanine	120	124	113	105	114
Arginine	56	61	63	55	53
Aspartate	50	53	48	35	45
Cysteine	0	0	0	1	0
Glycine	219	222	317	332	334
Glutamate	97	97	76	68	78
Histidine	11	7	0	5	6
Hydroxy lysine	13	10	0	12	9
Hydroxy proline	104	103	94	91	86
Isoleucine	13	15	11	12	10
Leucine	32	30	28	27	25
Lysine	26	21	30	27	24
Methionine	4	2	10	7	6
Phenylalanine	19	18	14	11	14
Proline	142	147	117	121	120
Serine	39	35	35	39	34
Threonine	22	20	18	21	17
Tryptophan	0	0	0	0	0
Tyrosine	5	2	4	3	4
Valine	28	27	23	26	26
*Imino acid*	246	250	211	212	205
TOT.	1000	1000	1000	1000	1000

**Table 2:** Marketed equine tendon collagen products sort by producer, form and application.

Company	Product	Additives	Form	Application	Ref.
B. & B. Dental (Bologna, Italy)	T-Barrier	-	Sheet	Hemostasis, Hard tissue	[103]
Baxter (Rome, Italy)	Gentafleece	Gentamicin sulphate	Sponge	Hemostasis, Wound healing	[104,105,106]
	TissuFoil E	-	Sheet	Wound healing	[107,108]
	TissuDura	-	Sheet	Wound healing	[109,110,111,112,113,114]
	TissueFleece	-	Sponge	Hemostasis	[115,116,117]
Zimmer Biomet (Warsaw, USA)	Septocoll	Gentamicin sulphate	Sponge	Hemostasis	[118]
Bioteck (Vicenza, Italy)	Biocollagen	-	Membrane	Hard tissues	[119,120,121]
	Bio-gen	Spongy bone	Powder	Hard tissues	[122]
	MeRG	Glycosaminoglycans	Membrane	Soft tissues	[123,124]
	Xenomatrix	-	Sheet	Soft tissues	[125,126]
Euroresearch (Milano, Italy)	Biopad	-	Sponge	Wound healing, Hard tissues	[26,127,128]
	Bioart	-	Powder	Hard tissues	-
	Nithya	-	Gel	Soft tissues, Anti-aging	[39]
	Revamil	Honey	Sponge	Wound healing	-
	Versuspray	Silver	Powder	Wound healing	-
EUSA Pharma (Langhorne, USA)	Collatamp	Gentamicin sulphate	Sponge	Wound healing	[129,130]
Finceramica (Faenza, Italy)	MaioRegen	Hydroxyapatite	Membrane	Soft tissue	[131,132,133,134]
Fidia Farmaceutici (Bologna, Italy)	Bionect pad	Hyaluronic acid	Sponge	Wound healing	[135]
Innocoll (Athlone, Ireland)	Collexa	Bovine collagen	Sponge	Wound healing	[136]
MLM Biologics (Gainesville, USA)	Bio-conneKt	-	Membrane	Wound healing	[136]
Nycomed (Munich, Germany)	TachoTop	-	Sponge	Hemostasis, Wound healing	[137,138]
	TachoComb	Human fibrinogen and bovine thrombin	Sponge	Hemostasis, Wound healing	[139,140,141,142,143]
	TachoSil	Human fibrinogen and human thrombin	Sponge	Hemostasis, Wound healing	[143,144,145,146,147,148,149,150,151,152]
Opocrin (Modena, Italy)	Antema	-	Sheet	Hemostasis, Wound healing	[57,153]
Resorba Medical GmbH (Nürnberg, Germany)	Genta-coll	Gentamicin sulphate	Sponge	Hemostasis, Hard tissues	[154,155,156]
	Kollagen	-	Sponge	Hemostasis, Hard tissues	[82,157,158,159,160,161,162,163,164,165,166]
	Parasorb	-	Membrane	Hemostasis, Hard tissues	[77,167,168]
Salvecoll (Como, Italy)	Salvecoll-E	-	Gel	Wound healing	[100]
Takeda (Tokyo, Japan)	CollGARA	-	Sponge	Hemostasis, Wound healing	[169]
GABA Vebas (Roma, Italy)	Paroguide	Chondroitin sulphate	Membrane	Wound healing	[170]

## Supplementary Materials

The following are available online at https://www.mdpi.com/2079-4983/11/4/79/s1, Figure S1: Amino acid sequences alignment of α1 and α2 chains of equine, bovine, suine, ovine, rodent and poultry collagen in comparison with human collagen, performed by mean of Clustal Omega (EMBL-EBI) sequence alignment bioinformatic tool.
