# Supplementary material for: An Overview of the Use of Equine Collagen as Emerging Material for Biomedical Applications
Source: J Funct Biomater. 2020 Nov 1;11(4):79. doi: 10.3390/jfb11040079 (PMC7712325; doi:10.3390/jfb11040079)
Supplement: Supplementary file 1 [file jfb-11-00079-s001.pdf]

Journal of *Functional Biomaterials*

Review

An Overview of the Use of Equine Collagen as Emerging Material for Biomedical Applications

Nunzia Gallo *, Maria Lucia Natali, Alessandro Sannino and Luca Salvatore

Department of Engineering for Innovation, University of Salento, Via Monteroni, 73100 Lecce, Italy; marialucia.natali@unisalento.it (M.L.N.); alessandro.sannino@unisalento.it (A.S.); luca.salvatore@unisalento.it (L.S.)
* Correspondence: nunzia.gallo@unisalento.it; Tel.: +39-0832-297236

Received: 8 October 2020; Accepted: 29 October 2020; Published: date

Figure S1. Amino acid sequences alignment of $\alpha1$ chains (**A**) and $\alpha2$ chains (**B**) of equine, bovine, swine, ovine, rodent and poultry collagen in comparison with human collagen performed by mean of the bioinformatic tool Clustal Omega (EMBL-EBI) (https://www.ebi.ac.uk/Tools/msa/clustalo/). Specie specific amino acid sequences of collagen were identified by mean of UniProtKB database (https://www.uniprot.org). Chains identifier are reported below. Asterisks highlight the conserved amino acids, while points those with the same chemical-physical characteristics.

(A)

```
SP|P02452|CO1A1_HUMAN        MFSFVDLRLLLLLAATALLTHGQEEGQVE---GQDEDIPPITCVQNGLRYHDRDVWKPEP 57

TR|F6SSG3|F6SSG3_HORSE       MFSFVDLRLLLLLAATALLTHGQEEGQEE---GQEEDIPAVTCIQDGLRYHDRAVWKPEP 57

SP|P02453|CO1A1_BOVIN        MFSFVDLRLLLLLAATALLTHGQEEGQEE---GQEEDIPPVTCVQNGLRYHDRDVWKPVP 57

TR|W5P481|W5P481_SHEEP       MFSFVDLRLLLLLAATALLTHGQEEGQEE---GQEEDIPPVTCVQNGLRYHDRDVWKPVP 57

TR|A0A287BLD2|A0A287BLD2_PIG MFSFVDLRLLLLLAATALLTHGQEEGQEEGQQGQEEDIPPVTCVQNGLRYHDRDVWKPVP 60
SP|P02454|CO1A1_RAT          MFSFVDLRLLLLLGATALLTHGQ------------EDIPEVSCIHNGLRVPNGETWKPDV 48
SP|P02457|CO1A1_CHICK        MFSFVDSRLLLLIAATVLLTRGEGE----------EDIQTGSCVQDGLTYNDKDVWKPEP 50
                             ****** *****:.**.***:*:            ***   :*:::**   :  .***
SP|P02452|CO1A1_HUMAN        CRICVCDNGKVLCDDVICDETKNCPGAEVPEGECCPVCPDGSESPTDQETTGVEGPKG-- 115
TR|F6SSG3|F6SSG3_HORSE       CRVCICDNGNVLCDDVICEDTKNCPGASVPKDECCPVCPEGQVSPTDDQTTGVEGPKG-- 115
SP|P02453|CO1A1_BOVIN        CQICVCDNGNVLCDDVICDELKDCPNAKVPTDECCPVCPEGQESPTDQETTGVEGPKG-- 115
TR|W5P481|W5P481_SHEEP       CQICVCDNGNVLCDDVICDELKDCPNAKVPTPPR-PVSPHPPPPPPPPTTTKQRGGRGGP 116
TR|A0A287BLD2|A0A287BLD2_PIG CQICVCDNGNVLCDDVICDEIKNCPSARVPAGECCPVCPEGEVSPTDQETTGVEGPKG-- 118
SP|P02454|CO1A1_RAT          CLICICHNGTAVCDGVLCKEDLDCPNPQKREGECCPFCPEEYVSP-DAEVIGVEGPKG-- 105
SP|P02457|CO1A1_CHICK        CQICVCDSGNILCDEVICEDTSDCPNAEIPFGECCPICPDVDASPVYPESAGVEGPKG-- 108
                             * :*:*..*. :** *:*.:  :**.         *..*.    *        .* :*
SP|P02452|CO1A1_HUMAN        -------DTGPRGPRGPAGPPGRDGIPGQPGLPGPPGPPGPPGPPGLGGNFAPQLSYGYD 168
TR|F6SSG3|F6SSG3_HORSE       -------DTGPRGPRGPAGPPGRDGIPGQPGLPGPPGPPGPPGPPGLGGNFAPQLSYGYD 168
SP|P02453|CO1A1_BOVIN        -------DTGPRGPRGPAGPPGRDGIPGQPGLPGPPGPPGPPGPPGLGGNFAPQLSYGYD 168
TR|W5P481|W5P481_SHEEP       CPRPGAHRPSHSPPQGPAGPPGRDGIPGQPGLPGPPGPPGPPGPPGLGGNFAPQLSYGYD 176
TR|A0A287BLD2|A0A287BLD2_PIG -------DTGPRGPRGPSGPPGRDGIPGQPGLPGPPGPPGPPGPPGLGGNFAPQLSYGYD 171
SP|P02454|CO1A1_RAT          -------DPGPQGPRGPVGPPGQDGIPGQPGLPGPPGPPGPPGPPGLGGNFASQMSYGYD 158
```

```
SP|P02457|CO1A1_CHICK        -------DTGPRGDRGLPGPPGRDGIPGQPGLPGPPG---PPGPPGLGGNFAPQMSYGYD 158
                                      .    :*  ****:**************   ************ *:*****
SP|P02452|CO1A1_HUMAN        EKSTGGISVPGPMGPSGPRGLPGPPGAPGPQGFQGPPGEPGEPGASGPMGPRGPPGPPGK 228
TR|F6SSG3|F6SSG3_HORSE       EKSA-GISVPGPMGPSGPRGLPGPPGAPGPQGFQGPPGEPGEPGASGPMGPRGPPGPPGK 227
SP|P02453|CO1A1_BOVIN        EKST-GISVPGPMGPSGPRGLPGPPGAPGPQGFQGPPGEPGEPGASGPMGPRGPPGPPGK 227
TR|W5P481|W5P481_SHEEP       EKST-GISVPGPMGPSGPRGLPGPPGAPGPQGFQGPPGEPGEPGASGPMGPRGPPGPPGK 235
TR|A0A287BLD2|A0A287BLD2_PIG EKSA-GISVPGPMGPSGPRGLPGPPGAPGPQGFQGPPGEPGEPGASGPMGPRGPPGPPGK 230
SP|P02454|CO1A1_RAT          EKSA-GVSVPGPMGPSGPRGLPGPPGAPGPQGFQGPPGEPGEPGASGPMGPRGPPGPPGK 217
SP|P02457|CO1A1_CHICK        EKSA-GVAVPGPMGPAGPRGLPGPPGAPGPQGFQGPPGEPGEPGASGPMGPRGPAGPPGK 217
                             ***: *::*******:************************************** *****
SP|P02452|CO1A1_HUMAN        NGDDGEAGKPGRPGERGPPGPQGARGLPGTAGLPGMKGHRGFSGLDGAKGDAGPAGPKGE 288
TR|F6SSG3|F6SSG3_HORSE       NGDDGEAGKPGRPGERGPPGPQGARGLPGTAGLPGMKGHRGFSGLDGAKGDAGPAGPKGE 287
SP|P02453|CO1A1_BOVIN        NGDDGEAGKPGRPGERGPPGPQGARGLPGTAGLPGMKGHRGFSGLDGAKGDAGPAGPKGE 287
TR|W5P481|W5P481_SHEEP       NGDDGEAGKPGRPGERGPPGPQGARGLPGTAGLPGMKGHRGFSGLDGAKGDAGPAGPKGE 295
TR|A0A287BLD2|A0A287BLD2_PIG NGDDGEAGKPGRPGERGPPGPQGARGLPGTAGLPGMKGHRGFSGLDGAKGDAGPAGPKGE 290
SP|P02454|CO1A1_RAT          NGDDGEAGKPGRPGERGPPGPQGARGLPGTAGLPGMKGHRGFSGLDGAKGDTGPAGPKGE 277
SP|P02457|CO1A1_CHICK        NGDDGEAGKPGRPGQRGPPGPQGARGLPGTAGLPGMKGHRGFSGLDGAKGQPGPAGPKGE 277
                             **************:***********************************: ********
SP|P02452|CO1A1_HUMAN        PGSPGENGAPGQMGPRGLPGERGRPGAPGPAGARGNDGATGAAGPPGPTGPAGPPGFPGA 348
TR|F6SSG3|F6SSG3_HORSE       PGSPGENGAPGQMGPRGLPGERGRPGAPGPAGARGNDGATGAAGPPGPTGPAGPPGFPGA 347
SP|P02453|CO1A1_BOVIN        PGSPGENGAPGQMGPRGLPGERGRPGAPGPAGARGNDGATGAAGPPGPTGPAGPPGFPGA 347
TR|W5P481|W5P481_SHEEP       PGSPGENGAPGQMGPRGLPGERGRPGAPGPAGARGNDGATGAAGPPGPTGPAGPPGFPGA 355
TR|A0A287BLD2|A0A287BLD2_PIG PGSPGENGAPGQMGPRGLPGERGRPGPPGPA---------------GPTGPAGPPGFPGA 335
SP|P02454|CO1A1_RAT          PGSPGENGAPGQMGPRGLPGERGRPGPPGSAGARGNDGAVGAAGPPGPTGPTGPPGFPGA 337
SP|P02457|CO1A1_CHICK        PGSPGENGAPGQMGPRGLPGERGRPGPSGPAGARGNDGAPGAAGPPGPTGPAGPPGFPGA 337
                             **************************  * *               *****:********
SP|P02452|CO1A1_HUMAN        VGAKGEAGPQGPRGSEGPQGVRGEPGPPGPAGAAGPAGNPGADGQPGAKGANGAPGIAGA 408
TR|F6SSG3|F6SSG3_HORSE       VGAKGEAGPQGARGSEGPQGVRGEPGPPGPAGAAGPAGNPGADGQPGAKGANGAPGIAGA 407
SP|P02453|CO1A1_BOVIN        VGAKGEGGPQGPRGSEGPQGVRGEPGPPGPAGAAGPAGNPGADGQPGAKGANGAPGIAGA 407
TR|W5P481|W5P481_SHEEP       VGAKGEAGPQGPRGSEGPQGVRGEPGPPGPAGAAGPAGNPGADGQPGAKGANGAPGIAGA 415
TR|A0A287BLD2|A0A287BLD2_PIG VGAKGEAGPQGARGSEGPQGVRGEPGPPGPAGAAGPAGNPGADGQPGGKGANGAPGIAGA 395
SP|P02454|CO1A1_RAT          AGAKGEAGPQGARGSEGPQGVRGEPGPPGPAGAAGPAGNPGADGQPGAKGANGAPGIAGA 397
SP|P02457|CO1A1_CHICK        AGAKGETGPQGARGSEGPQGSRGEPGPPGPAGAAGPAGNPGADGQPGAKGATGAPGIAGA 397
                             .***** **** ******** **************************.***.********
SP|P02452|CO1A1_HUMAN        PGFPGARGPSGPQGPGGPPGPKGNSGEPGAPGSKGDTGAKGEPGPVGVQGPPGPAGEEGK 468
TR|F6SSG3|F6SSG3_HORSE       PGFPGARGPSGPQGPSGPPGPKGNSGEPGAPGNKGDTGAKGEPGPTGIQGPPGPAGEEGK 467
SP|P02453|CO1A1_BOVIN        PGFPGARGPSGPQGPSGPPGPKGNSGEPGAPGSKGDTGAKGEPGPTGIQGPPGPAGEEGK 467
TR|W5P481|W5P481_SHEEP       PGFPGARGPSGPQGPSGPPGPKGNSGEPGAPGSKGDTGAKGEPGPTGIQGPPGPAGEEGK 475
TR|A0A287BLD2|A0A287BLD2_PIG PGFPGARGPSGPQGPSGPPGPKGNSGEPGAPGSKGDTGAKGEPGPTGVQGPPGPAGEEGK 455
SP|P02454|CO1A1_RAT          PGFPGARGPSGPQGPSGAPGPKGNSGEPGAPGNKGDTGAKGEPGPAGVQGPPGPAGEEGK 457
SP|P02457|CO1A1_CHICK        PGFPGARGPSGPQGPSGAPGPKGNSGEPGAPGNKGDTGAKGEPGPAGVQGPPGPAGEEGK 457
                             ***************.* **************.************.*:************
SP|P02452|CO1A1_HUMAN        RGARGEPGPTGLPGPPGERGGPGSRGFPGADGVAGPKGPAGERGSPGPAGPKGSPGEAGR 528
TR|F6SSG3|F6SSG3_HORSE       RGARGEPGPTGLPGPPGERGGPGARGFPGADGVAGPKGPAGERGAPGPAGPKGSPGEAGR 527
```

```
SP|P02453|CO1A1_BOVIN        RGARGEPGPAGLPGPPGERGGPGSRGFPGADGVAGPKGPAGERGAPGPAGPKGSPGEAGR 527
TR|W5P481|W5P481_SHEEP       RGARGEPGPAGLPGPPGERGGPGSRGFPGSDGVAGPKGPAGERGAPGPAGPKGSPGEAGR 535
TR|A0A287BLD2|A0A287BLD2_PIG RGARGEPGPAGLPGPPGERGGPGSRGFPGADGVAGPKGPAGERGSPGPAGPKGSPGEAGR 515
SP|P02454|CO1A1_RAT          RGARGEPGPSGLPGPPGERGGPGSRGFPGADGVAGPKGPAGERGSPGPAGPKGSPGEAGR 517
SP|P02457|CO1A1_CHICK        RGARGEPGPAGLPGPAGERGAPGSRGFPGADGIAGPKGPPGERGSPGAVGPKGSPGEAGR 517
                             *********:***** ****.**:*****:**:****** ****:** .***********
SP|P02452|CO1A1_HUMAN        PGEAGLPGAKGLTGSPGSPGPDGKTGPPGPAGQDGRPGPPGPPGARGQAGVMGFPGPKGA 588
TR|F6SSG3|F6SSG3_HORSE       PGEAGLPGAKGLTGSPGSPGPDGKTGPPGPAGQDGRPGPPGPPGARGQAGVMGFPGPKGA 587
SP|P02453|CO1A1_BOVIN        PGEAGLPGAKGLTGSPGSPGPDGKTGPPGPAGQDGRPGPPGPPGARGQAGVMGFPGPKGA 587
TR|W5P481|W5P481_SHEEP       PGEAGLPGAKGLTGSPGSPGPDGKTGPPGPAGQDGRPGPPGPPGARGQAGVMGFPGPKGA 595
TR|A0A287BLD2|A0A287BLD2_PIG PGEAGLPGAKGLTGSPGSPGPDGKTGPPGPAGQDGRPGPPGPPGARGQAGVMGFPGPKGA 575
SP|P02454|CO1A1_RAT          PGEAGLPGAKGLTGSPGSPGPDGKTGPPGPAGQDGRPGPAGPPGARGQAGVMGFPGPKGT 577
SP|P02457|CO1A1_CHICK        PGEAGLPGAKGLTGSPGSPGPDGKTGPPGPAGQDGRPGPAGPPGARGQAGVMGFPGPKGA 577
                             ************************************** ********************:
SP|P02452|CO1A1_HUMAN        AGEPGKAGERGVPGPPGAVGPAGKDGEAGAQGPPGPAGPAGERGEQGPAGSPGFQGLPGP 648
TR|F6SSG3|F6SSG3_HORSE       AGEPGKAGERGVPGPPGAVGPAGKDGEAGAQGPPGPAGPAGERGEQGPAGSPGFQGLPGP 647
SP|P02453|CO1A1_BOVIN        AGEPGKAGERGVPGPPGAVGPAGKDGEAGAQGPPGPAGPAGERGEQGPAGSPGFQGLPGP 647
TR|W5P481|W5P481_SHEEP       AGEPGKAGERGVPGPPGAVGPAGKDGEAGAQGPPGPAGPAGERGEQGPAGSPGFQGLPGP 655
TR|A0A287BLD2|A0A287BLD2_PIG AGEPGKAGERGVPGPPGAVGPAGKDGEAGAQGPPGPAGPAGERGEQGPAGSPGFQGLPGP 635
SP|P02454|CO1A1_RAT          AGEPGKAGERGVPGPPGAVGPAGKDGEAGAQGAPGPAGPAGERGEQGPAGSPGFQGLPGP 637
SP|P02457|CO1A1_CHICK        AGEPGKPGERGAPGPPGAVGAAGKDGEAGAQGPPGPTGPAGERGEQGPAGAPGFQGLPGP 637
                             ****** ****.******** *********** ***:*************:*********
SP|P02452|CO1A1_HUMAN        AGPPGEAGKPGEQGVPGDLGAPGPSGARGERGFPGERGVQGPPGPAGPRGANGAPGNDGA 708
TR|F6SSG3|F6SSG3_HORSE       AGPPGESGKPGEQGVPGDLGAPGPSGARGERGFPGERGVQGPPGPAGPRGSNGAPGNDGA 707
SP|P02453|CO1A1_BOVIN        AGPPGEAGKPGEQGVPGDLGAPGPSGARGERGFPGERGVQGPPGPAGPRGANGAPGNDGA 707
TR|W5P481|W5P481_SHEEP       AGPPGEAGKPGEQGVPGDLGAPGPSGARGERGFPGERGVQGPPGPAGPRGANGAPGNDGA 715
TR|A0A287BLD2|A0A287BLD2_PIG AGPPGEAGKPGEQGVPGDLGAPGPSGARGERGFPGERGVQGPPGPAGPRGANGAPGNDGA 695
SP|P02454|CO1A1_RAT          AGPPGEAGKPGEQGVPGDLGAPGPSGARGERGFPGERGVQGPPGPAGPRGNNGAPGNDGA 697
SP|P02457|CO1A1_CHICK        AGPPGEAGKPGEQGVPGNAGAPGPAGARGERGFPGERGVQGPPGPQGPRGANGAPGNDGA 697
                             ******:**********: *****:******************** **** *********
SP|P02452|CO1A1_HUMAN        KGDAGAPGAPGSQGAPGLQGMPGERGAAGLPGPKGDRGDAGPKGADGSPGKDGVRGLTGP 768
TR|F6SSG3|F6SSG3_HORSE       KGDAGAPGAPGSQGAPGLQGMPGERGAAGLPGPKGDRGDAGPKGADGSPGKDGVRGLTGP 767
SP|P02453|CO1A1_BOVIN        KGDAGAPGAPGSQGAPGLQGMPGERGAAGLPGPKGDRGDAGPKGADGAPGKDGVRGLTGP 767
TR|W5P481|W5P481_SHEEP       KGDAGAPGAPGSQGAPGLQGMPGERGAAGLPGPKGDRGDAGPKGADGAPGKDGVRGLTGP 775
TR|A0A287BLD2|A0A287BLD2_PIG KGDAGAPGAPGSQGAPGLQGMPGERGAAGLPGPKGDRGDAGPKGADGAPGKDGVRGLTGP 755
SP|P02454|CO1A1_RAT          KGDTGAPGAPGSQGAPGLQGMPGERGAAGLPGPKGDRGDAGPKGADGSPGKDGVRGLTGP 757
SP|P02457|CO1A1_CHICK        KGDAGAPGAPGNEGPPGLEGMPGERGAAGLPGAKGDRGDPGPKGADGAPGKDGLRGLTGP 757
                             ***:*******.:* ***:************* ****** *******:*****:******
SP|P02452|CO1A1_HUMAN        IGPPGPAGAPGDKGESGPSGPAGPTGARGAPGDRGEPGPPGPAGFAGPPGADGQPGAKGE 828
TR|F6SSG3|F6SSG3_HORSE       IGPPGPAGAPGDKGETGPSGPAGPTGARGAPGDRGEPGPPGPAGFAGPPGADGQPGAKGE 827
SP|P02453|CO1A1_BOVIN        IGPPGPAGAPGDKGEAGPSGPAGPTGARGAPGDRGEPGPPGPAGFAGPPGADGQPGAKGE 827
TR|W5P481|W5P481_SHEEP       IGPPGPAGAPGDKGETGPSGPAGPTGARGAPGDRGEPGPPGPAGFAGPPGADGQPGAKGE 835
TR|A0A287BLD2|A0A287BLD2_PIG IGPPGPAGAPGDKGETGPSGPAGPTGARGAPGDRGEPGPPGPAGFAGPPGADGQPGAKGE 815
SP|P02454|CO1A1_RAT          IGPPGPAGAPGDKGETGPSGPAGPTGARGAPGDRGEPGPPGPAGFAGPPGADGQPGAKGE 817
```

```
SP|P02457|CO1A1_CHICK        IGPPGPAGAPGDKGEAGPPGPAGPTGARGAPGDRGEPGPPGPAGFAGPPGADGQPGAKGE 817
                             ***************:** ****************************************
SP|P02452|CO1A1_HUMAN        PGDAGAKGDAGPPGPAGPAGPPGPIGNVGAPGAKGARGSAGPPGATGFPGAAGRVGPPGP 888
TR|F6SSG3|F6SSG3_HORSE       PGDAGAKGDAGPPGPAGPAGPPGPIGSVGAPGPKGARGSAGPPGATGFPGAAGRVGPPGP 887
SP|P02453|CO1A1_BOVIN        PGDAGAKGDAGPPGPAGPAGPPGPIGNVGAPGPKGARGSAGPPGATGFPGAAGRVGPPGP 887
TR|W5P481|W5P481_SHEEP       PGDAGAKGDAGPPGPAGPAGPPGPIGNVGAPGPKGARGSAGPPGATGFPGAAGRVGPPGP 895
TR|A0A287BLD2|A0A287BLD2_PIG PGDAGAKGDAGPPGPAGPTGPPGPIGSVGAPGPKGARGSAGPPGATGFPGAAGRVGPPGP 875
SP|P02454|CO1A1_RAT          PGDTGVKGDAGPPGPAGPAGPPGPIGNVGAPGPKGSRGAAGPPGATGFPGAAGRVGPPGP 877
SP|P02457|CO1A1_CHICK        TGDAGAKGDAGPPGPAGPTGAPGPAGZVGAPGPKGARGSAGPPGATGFPGAAGRVGPPGP 877
                              **:*.************:* *** * ***** **:**:*********************
SP|P02452|CO1A1_HUMAN        SGNAGPPGPPGPAGKEGGKGPRGETGPAGRPGEVGPPGPPGPAGEKGSPGADGPAGAPGT 948
TR|F6SSG3|F6SSG3_HORSE       SGNAGPPGPPGPVGKEGGKGPRGETGPAGRPGEAGPPGPPGPAGEKGSPGADGPAGAPGT 947
SP|P02453|CO1A1_BOVIN        SGNAGPPGPPGPAGKEGSKGPRGETGPAGRPGEVGPPGPPGPAGEKGAPGADGPAGAPGT 947
TR|W5P481|W5P481_SHEEP       SGNAGPPGPPGPAGKEGSKGPRGETGPAGRAGEVGPPGPPGPAGEKGAPGADGPAGAPGT 955
TR|A0A287BLD2|A0A287BLD2_PIG SGNAGPPGPPGPAGKEGSKGPRGETGPAGRPGEAGPPGPPGPAGEKGSPGADGPAGAPGT 935
SP|P02454|CO1A1_RAT          SGNAGPPGPPGPVGKEGGKGPRGETGPAGRPGEVGPPGPPGPAGEKGSPGADGPAGSPGT 937
SP|P02457|CO1A1_CHICK        SGNIGLPGPPGPAGKZGSKGPRGETGPAGRPGEPGPAGPPGPPGEKGSPGADGPIGAPGT 937
                             *** * ******.** *.************ ** ** ***** ****:****** *:***
SP|P02452|CO1A1_HUMAN        PGPQGIAGQRGVVGLPGQRGERGFPGLPGPSGEPGKQGPSGASGERGPPGPMGPPGLAGP 1008
TR|F6SSG3|F6SSG3_HORSE       PGPQGIAGQRGVVGLPGQRGERGFPGLPGPSGEPGKQGPSGASGERGPPGPVGPPGLAGP 1007
SP|P02453|CO1A1_BOVIN        PGPQGIAGQRGVVGLPGQRGERGFPGLPGPSGEPGKQGPSGASGERGPPGPMGPPGLAGP 1007
TR|W5P481|W5P481_SHEEP       PGPQGIAGQRGVVGLPGQRGERGFPGLPGPSGEPGKQGPSGASGERGPPGPMGPPGLAGP 1015
TR|A0A287BLD2|A0A287BLD2_PIG PGPQGIAGQRGVVGLPGQRGERGFPGLPGPSGEPGKQGPSGPSGERGPPGPMGPPGLAGP 995
SP|P02454|CO1A1_RAT          PGPQGIAGQRGVVGLPGQRGERGFPGLPGPSGEPGKQGPSGASGERGPPGPMGPPGLAGP 997
SP|P02457|CO1A1_CHICK        PGPQGIAGQRGVVGLPGQRGERGFPGLPGPSGEPGKQGPSGASGERGPPGPMGPPGLAGP 997
                             ***************************************** *********:********
SP|P02452|CO1A1_HUMAN        PGESGREGAPGAEGSPGRDGSPGAKGDRGETGPAGPPGAPGAPGAPGPVGPAGKSGDRGE 1068
TR|F6SSG3|F6SSG3_HORSE       PGESGREGSPGAEGSPGRDGSPGPKGDRGETGPAGPPGAPGAPGAPGPVGPAGKSGDRGE 1067
SP|P02453|CO1A1_BOVIN        PGESGREGAPGAEGSPGRDGSPGAKGDRGETGPAGPPGAPGAPGAPGPVGPAGKSGDRGE 1067
TR|W5P481|W5P481_SHEEP       PGESGREGAPGAEGSPGRDGAPGAKGDRGETGPAGPPGAPGAPGAPGPVGPAGKSGDRGE 1075
TR|A0A287BLD2|A0A287BLD2_PIG PGESGREGAPGAEGSPGRDGAPGPKGDRGESGPAGPPGAPGAPGAPGPVGPAGKSGDRGE 1055
SP|P02454|CO1A1_RAT          PGESGREGSPGAEGSPGRDGAPGAKGDRGETGPAGPPGAPGAPGAPGPVGPAGKNGDRGE 1057
SP|P02457|CO1A1_CHICK        PGEAGREGAPGAEGAPGRDGAAGPKGDRGETGPAGPPGAPGAPGAPGPVGPAGKNGDRGE 1057
                             ***:****:*****:*****: * ******:**********************.*****
SP|P02452|CO1A1_HUMAN        TGPAGPTGPVGPVGARGPAGPQGPRGDKGETGEQGDRGIKGHRGFSGLQGPPGPPGSPGE 1128
TR|F6SSG3|F6SSG3_HORSE       AGPAGPAGPIGPVGARGPAGPQGPRGDKGETGEQGDRGIKGHRGFSGLQGPPGPPGSPGE 1127
SP|P02453|CO1A1_BOVIN        TGPAGPAGPIGPVGARGPAGPQGPRGDKGETGEQGDRGIKGHRGFSGLQGPPGPPGSPGE 1127
TR|W5P481|W5P481_SHEEP       TGPAGPAGPIGPVGARGPAGPQGPRGDKGETGEQGDRGIKGHRGFSGLQGPPGPPVSMLS 1135
TR|A0A287BLD2|A0A287BLD2_PIG TGPAGPAGPVGPVGARGPAGPQGPRGDKGETGEQGDRGIKGHRGFSGLQGPPGPPGSPGE 1115
SP|P02454|CO1A1_RAT          TGPAGPAGPIGPAGARGPAGPQGPRGDKGETGEQGDRGIKGHRGFSGLQGPPGSPGSPGE 1117
SP|P02457|CO1A1_CHICK        TGPAGPAGPPGPAGARGPAGPQGPRGDKGETGEQGDRGMKGHRGFSGLQGPPGPPGAPGE 1117
                             :*****:** **.*************************:************* * :  .
SP|P02452|CO1A1_HUMAN        QGPSGASGPAGPRGPPGSAGAPGKDGLNGLPGPIGPPGPRGRTGDAGPVGPPGPPGPPGP 1188
TR|F6SSG3|F6SSG3_HORSE       QGPSGASGPAGPRGPPGSAGAPGKDGLNGLPGPIGPPGPRGRTGDAGPVGPPGPPGPPGP 1187
```

```
SP|P02453|CO1A1_BOVIN        QGPSGASGPAGPRGPPGSAGSPGKDGLNGLPGPIGPPGPRGRTGDAGPAGPPGPPGPPGP 1187
TR|W5P481|W5P481_SHEEP       PSPAP-SPASSLQGPPGSAGTPGKDGLNGLPGPIGPPGPRGRTGDAGPAVSPPTLSAHGP 1194
TR|A0A287BLD2|A0A287BLD2_PIG QGPSGASGPAGPRGPPGSAGAPGKDGLNGLPGPIGPPGPRGRTGDAGPVGPPGPPGPPGP 1175
SP|P02454|CO1A1_RAT          QGPSGASGPAGPRGPPGSAGSPGKDGLNGLPGPIGPPGPRGRTGDSGPAGPPGPPGPPGP 1177
SP|P02457|CO1A1_CHICK        QGPSGASGPAGPRGPPGSAGAAGKDGLNGLPGPIGPPGPRGRTGEVGPVGPPGPPGPPGP 1177
                              .*:  *  :. :*******: **********************: **.  *   .  **
SP|P02452|CO1A1_HUMAN        P----GPPSAGFDFSFLPQPPQEKAHDGGRYYRADDANVVRDRDLEVDTTLKSLSQQIEN 1244
TR|F6SSG3|F6SSG3_HORSE       P----GPPSGGFDFSFLPQPPQEKSHDGGRYYRADDANVVRDRDLEVDTTLKSLSQQIEN 1243
SP|P02453|CO1A1_BOVIN        P----GPPSGGYDLSFLPQPPQEKAHDGGRYYRADDANVVRDRDLEVDTTLKSLSQQIEN 1243
TR|W5P481|W5P481_SHEEP       PALKAPSPRSRYDLSFLPQPPQEKAHDGGRYYRADDANVVRDRDLEVDTTLKSLSQQIEN 1254
TR|A0A287BLD2|A0A287BLD2_PIG P----GPPSGGFDFSFLPQPPQEKAHDGGRYYRADDANVVRDRDLEVDTTLKSLSQQIEN 1231
SP|P02454|CO1A1_RAT          P----GPPSGGYDFSFLPQPPQEKSQDGGRYYRADDANVVRDRDLEVDTTLKSLSQQIEN 1233
SP|P02457|CO1A1_CHICK        P----GPPSGGFDLSFLPQPPQEKAHDGGRYYRADDANVMRDRDLEVDTTLKSLSQQIEN 1233
                             *      * . :*:**********::*************:********************
SP|P02452|CO1A1_HUMAN        IRSPEGSRKNPARTCRDLKMCHSDWKSGEYWIDPNQGCNLDAIKVFCNMETGETCVYPTQ 1304
TR|F6SSG3|F6SSG3_HORSE       IRSPEGSRKNPARTCRDLKMCHSDWKSGEYWIDPNQGCNLDAIKVFCNMETGETCVYPTQ 1303
SP|P02453|CO1A1_BOVIN        IRSPEGSRKNPARTCRDLKMCHSDWKSGEYWIDPNQGCNLDAIKVFCNMETGETCVYPTQ 1303
TR|W5P481|W5P481_SHEEP       IRSPEGSRKNPARTCRDLKMCHPDWKSGEYWIDPNQGCNLDAIKVFCNMETGETCVYPTQ 1314
TR|A0A287BLD2|A0A287BLD2_PIG IRSPEGSRKNPARTCRDLKMCHSDWKSGEYWIDPNQGCNLDAIKVFCNMETGETCVYPTQ 1291
SP|P02454|CO1A1_RAT          IRSPEGSRKNPARTCRDLKMCHSDWKSGEYWIDPNQGCNLDAIKVYCNMETGQTCVFPTQ 1293
SP|P02457|CO1A1_CHICK        IRSPEGTRKNPARTCRDLKMCHGDWKSGEYWIDPNQGCNLDAIKVYCNMETGETCVYPTQ 1293
                             ******:*************** **********************:******:***:***
SP|P02452|CO1A1_HUMAN        PSVAQKNWYISKNPKDKRHVWFGESMTDGFQFEYGGQGSDPADVAIQLTFLRLMSTEASQ 1364
TR|F6SSG3|F6SSG3_HORSE       PQVAQKNWYISKNPKDKRHVWYGESMTDGFQFEYGGQGSDPADVAIQLTFLRLMSTEASQ 1363
SP|P02453|CO1A1_BOVIN        PSVAQKNWYISKNPKEKRHVWYGESMTGGFQFEYGGQGSDPADVAIQLTFLRLMSTEASQ 1363
TR|W5P481|W5P481_SHEEP       PSVPQKNWYISKNPKDKRHVWYGESMTGGFQVREGGQGSDPADVAIQLTFLRLMSTEASQ 1374
TR|A0A287BLD2|A0A287BLD2_PIG PSVPQKNWYISKNPKDKRHVWYGESMTDGFQFEYGGEGSDPADVAIQLTFLRLMSTEASQ 1351
SP|P02454|CO1A1_RAT          PSVPQKNWYISPNPKEKKHVWFGESMTDGFQFEYGSEGSDPADVAIQLTFLRLMSTEASQ 1353
SP|P02457|CO1A1_CHICK        ATIAQKNWYLSKNPKEKKHVWFGETMSDGFQFEYGGEGSNPADVAIQLTFLRLMSTEATQ 1353
                               : *****:* ***:*:***:**:*:.***.. *.:**:******************:*
SP|P02452|CO1A1_HUMAN        NITYHCKNSVAYMDQQTGNLKKALLLQGSNEIEIRAEGNSRFTYSVTVDGCTSHTGAWGK 1424
TR|F6SSG3|F6SSG3_HORSE       NITYHCKNSVAYMDQQTGNLKKALLLQGSNEIEIRAEGNSRFTYSVTYDGCTSHTGAWGK 1423
SP|P02453|CO1A1_BOVIN        NITYHCKNSVAYMDQQTGNLKKALLLQGSNEIEIRAEGNSRFTYSVTYDGCTSHTGAWGK 1423
TR|W5P481|W5P481_SHEEP       NITYHCKNSVAYMDQQTGNLKKALLLQGSNEIEIRAEGNSRFTYSVTYDGCTSHTGAWGK 1434
TR|A0A287BLD2|A0A287BLD2_PIG NITYHCKNSVAYMDQQTGNLKKALLLQGSNEIEIRAEGNSRFTYSVIYDGCTSHTGAWGK 1411
SP|P02454|CO1A1_RAT          NITYHCKNSVAYMDQQTGNLKKSLLLQGSNEIELRGEGNSRFTYSTLVDGCTSHTGTWGK 1413
SP|P02457|CO1A1_CHICK        NVTYHCKNSVAYMDHDTGNLKKALLLQGANEIEIRAEGNSRFTYGVTEDGCTSHTGAWGK 1413
                             *:************::******:*****:****:*.********..  ********:***
SP|P02452|CO1A1_HUMAN        TVIEYKTTKTSRLPIIDVAPLDVGAPDQEFGFDVGPVCFL 1464
TR|F6SSG3|F6SSG3_HORSE       TVIEYKTTKTSRLPIIDVAPLDIGAPDQEFGIDIGPVCFL 1463
SP|P02453|CO1A1_BOVIN        TVIEYKTTKTSRLPIIDVAPLDVGAPDQEFGFDVGPACFL 1463
TR|W5P481|W5P481_SHEEP       TVIEYKTTKTSRLPIIDVAPLDVGAPDQEFGFDIGSVCFL 1474
TR|A0A287BLD2|A0A287BLD2_PIG TVIEYKTTKTSRLPIIDVAPLDVGAPDQEFGIDLSPVCFL 1451
SP|P02454|CO1A1_RAT          TVIEYKTTKTSRLPIIDVAPLDIGAPDQEFGMDIGPACFV 1453
```

```
SP|P02457|CO1A1_CHICK        TVIEYKTTKTSRLPIIDLAPMDVGAPDQEFGIDIGPVCFL 1453
                             ****************:**:*:********:*:. .**:
```

(B)

```
SP|P08123|CO1A2_HUMAN        MLSFVDTRTLLLLAVTLCLATCQSLQE-ETVRKGPAGDRGPRGERGPPGPPGRDGEDGPT 59
TR|F6RTI8|F6RTI8_HORSE       MLSFVDTRTLLLLAVTSCLATCQSLQE-ATAGKGPTGDRGPRGERGPPGPPGRDGDDGIP 59
SP|P02465|CO1A2_BOVIN        MLSFVDTRTLLLLAVTSCLATCQSLQE-ATARKGPSGDRGPRGERGPPGPPGRDGDDGIP 59
TR|W5NTT7|W5NTT7_SHEEP       MLSFVDTRTLLLLAVTSCLATCQSLQE-ATARKGPSGDRGPRGERGPPGPPGRDGDDGIP 59
TR|A0A1S7J1Y9|A0A1S7J1Y9_PIG MLSFVDTRTLLLLAVTSCLATCQSLQE-ATARKGPTGDRGPRGERGPPGPPGRDGDDGIP 59
SP|P02466|CO1A2_RAT          MLSFVDTRTLLLLAVTSCLATCQSLQM-GSVRKGPTGDRGPRGQRGPAGPRGRDGVDGPV 59
SP|P02467|CO1A2_CHICK        MLSFVDTRILLLLAVTSYLATSQHLFQASAGRKGPRGDKGPQGERGPPGPPGRDGEDGPP 60
                             ******** *******  ***.* *    :  *** **:**:*:*** ** **** **
SP|P08123|CO1A2_HUMAN        GPPGP------PGPPGPPGLGGNFAAQYDGK-GVGLGPGPMGLMGPRGPPGAAGAPGPQG 112
TR|F6RTI8|F6RTI8_HORSE       GPPGP------PGPPGPPGLGGNFAAQFDAK-G--GGPGPMGLMGPRGPPGASGAPGPQG 110
SP|P02465|CO1A2_BOVIN        GPPGP------PGPPGPPGLGGNFAAQFDAK-G--GGPGPMGLMGPRGPPGASGAPGPQG 110
TR|W5NTT7|W5NTT7_SHEEP       GPPGP------PGPPGPPGLGGNFAAQFDGK-G--GGPGPMGLMGPRGPPGASGAPGPQG 110
TR|A0A1S7J1Y9|A0A1S7J1Y9_PIG GPPGP------PGPPGPPGLGGNFAAQYDGK-GVGAGPGPMGLMGPRGPPGAVGAPGPQG 112
SP|P02466|CO1A2_RAT          GPPGPPGAPGPPGPPGPPGLTGNFAAQYSDK-GVSAGPGPMGLMGPRGPPGAVGAPGPQG 118
SP|P02467|CO1A2_CHICK        GP---------PGPPGPPGLGGNFAAQYDPSKAADFGPGPMGLMGPRGPPGASGPPGPPG 111
                             **         ********* ******:. . .   *************** * *** *
SP|P08123|CO1A2_HUMAN        FQGPAGEPGEPGQTGPAGARGPAGPPGKAGEDGHPGKPGRPGERGVVGPQGARGFPGTPG 172
TR|F6RTI8|F6RTI8_HORSE       FQGPAGEPGEPGQTGPAGARGPPGPPGKAGEDGHPGKPGRPGERGVVGPQGARGFPGTPG 170
SP|P02465|CO1A2_BOVIN        FQGPPGEPGEPGQTGPAGARGPPGPPGKAGEDGHPGKPGRPGERGVVGPQGARGFPGTPG 170
TR|W5NTT7|W5NTT7_SHEEP       FQGPPGEPGEPGQTGPAGARGPPGPPGKAGEDGHPGKPGRPGERGVVGPQGARGFPGTPG 170
TR|A0A1S7J1Y9|A0A1S7J1Y9_PIG FQGPAGEPGEPGQTGPAGARGPPGPPGKAGEDGHPGKPGRPGERGVVGPQGARGFPGTPG 172
SP|P02466|CO1A2_RAT          FQGPAGEPGEPGQTGPAGSRGPAGPPGKAGEDGHPGKPGRPGERGVVGPQGARGFPGTPG 178
SP|P02467|CO1A2_CHICK        FQGVPGEPGEPGQTGPQGPRGPPGPPGKAGEDGHPGKPGRPGERGVAGPQGARGFPGTPG 171
                             ***  *********** * *** *********************.*************
SP|P08123|CO1A2_HUMAN        LPGFKGIRGHNGLDGLKGQPGAPGVKGEPGAPGENGTPGQTGARGLPGERGRVGAPGPAG 232
TR|F6RTI8|F6RTI8_HORSE       LPGFKGIRGHNGLDGLKGQPGAPGVKGEPGAPGENGTPGQAGARGLPGERGRVGAPGPAG 230
SP|P02465|CO1A2_BOVIN        LPGFKGIRGHNGLDGLKGQPGAPGVKGEPGAPGENGTPGQTGARGLPGERGRVGAPGPAG 230
TR|W5NTT7|W5NTT7_SHEEP       LPGFKGIRGHNGLDGLKGQPGAPGVKGEPGAPGENGTPGQTGARGLPGERGRVGAPGPAG 230
TR|A0A1S7J1Y9|A0A1S7J1Y9_PIG LPGFKGIRGHNGLDGLKGQPGAPGVKGEPGAPGENGTPGQTGARGLPGERGRVGAPGPAG 232
SP|P02466|CO1A2_RAT          LPGFKGIRGHNGLDGLKGQPGAQGVKGEPGAPGENGTPGQAGARGLPGERGRVGAPGPAG 238
SP|P02467|CO1A2_CHICK        LPGFKGIRGHNGLDGQKGQPGTPGTKGEPGAPGENGTPGQPGARGLPGERGRIGAPGPAG 231
                             *************** *****: *.*************** **********:*******
SP|P08123|CO1A2_HUMAN        ARGSDGSVGPVGPAGPIGSAGPPGFPGAPGPKGEIGAVGNAGPAGPAGPRGEVGLPGLSG 292
TR|F6RTI8|F6RTI8_HORSE       ARGSDGSVGPVGPAGPIGSAGPPGFPGAPGPKGELGPVGNPGPAGPAGPRGEVGLPGLSG 290
SP|P02465|CO1A2_BOVIN        ARGSDGSVGPVGPAGPIGSAGPPGFPGAPGPKGELGPVGNPGPAGPAGPRGEVGLPGLSG 290
TR|W5NTT7|W5NTT7_SHEEP       ARGSDGSVGPVGPAGPIGSAGPPGFPGAPGPKGELGPVGNPGPAGPAGPRGEVGLPGLSG 290
TR|A0A1S7J1Y9|A0A1S7J1Y9_PIG ARGNDGSVGPVGPAGPIGSAGPPGFPGAPGPKGELGPVGNPGPAGPAGPRGEVGLPGVSG 292
SP|P02466|CO1A2_RAT          ARGSDGSVGPVGPAGPIGSAGPPGFPGAPGPKGELGPVGNPGPAGPAGPRGEAGLPGLSG 298
SP|P02467|CO1A2_CHICK        ARGSDGSAGPTGPAGPIGAAGPPGFPGAPGAKGEIGPAGNVGPTGPAGPRGEIGLPGSSG 291
                             ***.***.**.*******:********** ***:* .** **:******** **** **
```

```
SP|P08123|CO1A2_HUMAN         PVGPPGNPGANGLTGAKGAAGLPGVAGAPGLPGPRGIPGPVGAAGATGARGLVGEPGPAG 352
TR|F6RTI8|F6RTI8_HORSE        PVGPPGNPGANGLTGAKGAAGLPGVAGAPGLPGPRGIPGPAGAAGATGARGLVGEPGPAG 350
SP|P02465|CO1A2_BOVIN         PVGPPGNPGANGLPGAKGAAGLPGVAGAPGLPGPRGIPGPVGAAGATGARGLVGEPGPAG 350
TR|W5NTT7|W5NTT7_SHEEP        PVGPPGNPGANGLPGAKGAAGLPGVAGAPGLPGPRGIPGPVGAAGATGARGLVGEPGPAG 350
TR|A0A1S7J1Y9|A0A1S7J1Y9_PIG  PVGPPGNPGANGLPGAKGAAGLPGVAGAPGLPGPRGIPGPAGAAGATGARGLVGEPGPAG 352
SP|P02466|CO1A2_RAT           PVGPPGNPGANGLTGAKGATGLPGVAGAPGLPGPRGIPGPVGAAGATGPRGLVGEPGPAG 358
SP|P02467|CO1A2_CHICK         PVGPPGNPGANGLPGAKGAAGLPGVAGAPGLPGPRGIPGPPGPAGPSGARGLVGEPGPAG 351
                              ************* *****:******************** * ** :* ***********
SP|P08123|CO1A2_HUMAN         SKGESGNKGEPGSAGPQGPPGPSGEEGKRGPNGEAGSAGPPGPPGLRGSPGSRGLPGADG 412
TR|F6RTI8|F6RTI8_HORSE        SKGESGNKGEPGAAGPQGPPGPSGEEGKRGPNGEPGSTGPAGPPGLRGSPGSRGLPGADG 410
SP|P02465|CO1A2_BOVIN         SKGESGNKGEPGAVGQPGPPGPSGEEGKRGSTGEIGPAGPPGPPGLRGNPGSRGLPGADG 410
TR|W5NTT7|W5NTT7_SHEEP        SKGESGNKGEPGAVGQPGPPGPSGEEGKRGSTGEIGPAGPPGPPGLRGNPGSRGLPGADG 410
TR|A0A1S7J1Y9|A0A1S7J1Y9_PIG  SKGESGNKGEPGAAGPQGPPGPSGEEGKRGPNGEVGSAGPPGPPGLRGNPGSRGLPGADG 412
SP|P02466|CO1A2_RAT           SKGETGNKGEPGSAGAQGPPGPSGEEGKRGSPGEPGSAGPAGPPGLRGSPGSRGLPGADG 418
SP|P02467|CO1A2_CHICK         AKGESGNKGEPGAAGPPGPPGPSGEEGKRGSNGEPGSAGPPGPAGLRGVPGSRGLPGADG 411
                              :***:*******:.*  *************  ** * :** ** **** ***********
SP|P08123|CO1A2_HUMAN         RAGVMGPPGSRGASGPAGVRGPNGDAGRPGEPGLMGPRGLPGSPGNIGPAGKEGPVGLPG 472
TR|F6RTI8|F6RTI8_HORSE        RAGVMGPAGSRGASGPAGVRGPNGDSGRPGEPGLMGPRGFPGSPGNIGPAGKEGPVGLPG 470
SP|P02465|CO1A2_BOVIN         RAGVMGPAGSRGATGPAGVRGPNGDSGRPGEPGLMGPRGFPGSPGNIGPAGKEGPVGLPG 470
TR|W5NTT7|W5NTT7_SHEEP        RAGVMGPAGSRGATGPAGVRGPNGDSGRPGEPGLMGPRGFPGSPGNIGPAGKEGPAGLPG 470
TR|A0A1S7J1Y9|A0A1S7J1Y9_PIG  RAGVMGPPGSRGPTGPAGVRGPNGDSGRPGEPGLMGPRGFPGSPGNVGPAGKEGPAGLPG 472
SP|P02466|CO1A2_RAT           RAGVMGPPGNRGSTGPAGVRGPNGDAGRPGEPGLMGPRGLPGSPGNVGPAGKEGPVGLPG 478
SP|P02467|CO1A2_CHICK         RAGVMGPAGNRGASGPVGAKGPNGDAGRPGEPGLMGPRGLPGQPGSPGPAGKEGPVGFPG 471
                              ******* *.** :**.*.:*****:*************:**.**. ********.*:**
SP|P08123|CO1A2_HUMAN         IDGRPGPIGPAGARGEPGNIGFPGPKGPTGDPGKNGDKGHAGLAGARGAPGPDGNNGAQG 532
TR|F6RTI8|F6RTI8_HORSE        IDGRPGPIGPAGARGEPGNIGFPGPKGPSGEPGKPGDKGHAGLAGARGAPGPDGNNGAQG 530
SP|P02465|CO1A2_BOVIN         IDGRPGPIGPAGARGEPGNIGFPGPKGPSGDPGKAGEKGHAGLAGARGAPGPDGNNGAQG 530
TR|W5NTT7|W5NTT7_SHEEP        IDGRPGPIGPAGARGEPGNIGFPGPKGPTGDPGKAGEKGHAGLAGPRGAPGPDGNNGAQG 530
TR|A0A1S7J1Y9|A0A1S7J1Y9_PIG  IDGRPGPIGPAGARGEPGNIGFPGPKGPTGDPGKNGEKGHAGLAGARGAPGPDGNNGAQG 532
SP|P02466|CO1A2_RAT           IDGRPGPIGPAGPRGEAGNIGFPGPKGPSGDPGKPGEKGHPGLAGARGAPGPDGNNGAQG 538
SP|P02467|CO1A2_CHICK         ADGRVGPIGPAGNRGEPGNIGFPGPKGPTGEPGKPGEKGNVGLAGPRGAPGPEGNNGAQG 531
                               *** ******* *** ***********:*:*** *:**: **** ******:*******
SP|P08123|CO1A2_HUMAN         PPGPQGVQGGKGEQGPPGPPGFQGLPGPSGPAGEVGKPGERGLHGEFGLPGPAGPRGERG 592
TR|F6RTI8|F6RTI8_HORSE        PPGPQGVQGGKGEQGPAGPPGFQGLPGPAGTAGEVGKPGERGLPGEFGLPGPAGARGERG 590
SP|P02465|CO1A2_BOVIN         PPGLQGVQGGKGEQGPAGPPGFQGLPGPAGTAGEAGKPGERGIPGEFGLPGPAGARGERG 590
TR|W5NTT7|W5NTT7_SHEEP        PPGLQGVQGGKGEQGPAGPPGFQGLPGPAGTAGEAGKPGERGIPGEFGLPGPAGARGERG 590
TR|A0A1S7J1Y9|A0A1S7J1Y9_PIG  PPGPQGVQGGKGEQGPAGPPGFQGLPGPAGTAGEVGKPGERGIPGEFGLPGPAGPRGERG 592
SP|P02466|CO1A2_RAT           PPGPQGVQGGKGEQGPAGPPGFQGLPGPSGTAGEVGKPGERGLPGEFGLPGPAGPRGERG 598
SP|P02467|CO1A2_CHICK         PPGVTGNQGAKGETGPAGPPGFQGLPGPSGPAGEAGKPGERGLHGEFGVPGPAGPRGERG 591
                              ***  * **.*** ** ***********:* ***.*******: ****:***** *****
SP|P08123|CO1A2_HUMAN         PPGESGAAGPTGPIGSRGPSGPPGPDGNKGEPGVVGAVGTAGPSGPSGLPGERGAAGIPG 652
TR|F6RTI8|F6RTI8_HORSE        PPGESGAAGPAGPIGSRGPSGPPGPDGNKGEPGVLGAPGTAGPSGPSGLPGERGAAGIPG 650
SP|P02465|CO1A2_BOVIN         PPGESGAAGPTGPIGSRGPSGPPGPDGNKGEPGVVGAPGTAGPSGPSGLPGERGAAGIPG 650
TR|W5NTT7|W5NTT7_SHEEP        PPGESGAAGPTGPIGSRGPSGPPGPDGNKGEPGVVGAPGTAGPSGPSGLPGERGAAGIPG 650
```

```
TR|A0A1S7J1Y9|A0A1S7J1Y9_PIG PPGESGAAGPAGPIGSRGPSGPPGPDGNKGEPGVLGAPGTAGPSGPSGLPGERGAAGIPG 652
SP|P02466|CO1A2_RAT          PPGESGAAGPSGPIGIRGPSGAPGPDGNKGEAGAVGAPGSAGASGPGGLPGERGAAGIPG 658
SP|P02467|CO1A2_CHICK        LPGESGAVGPAGPIGSRGPSGPPGPDGNKGEPGNVGPAGAPGPAGPGGIPGERGVAGVPG 651
                              ******.**:**** ***** ********* * :*  *: * :**.*:*****.**:**

SP|P08123|CO1A2_HUMAN        GKGEKGEPGLRGEIGNPGRDGARGAPGAVGAPGPAGATGDRGEAGAAGPAGPAGPRGSPG 712
TR|F6RTI8|F6RTI8_HORSE       GKGEKGETGLRGEIGNPGRDGARGAPGAVGAPGPAGANGDRGEAGAAGPAGPAGPRGSPG 710
SP|P02465|CO1A2_BOVIN        GKGEKGETGLRGDIGSPGRDGARGAPGAIGAPGPAGANGDRGEAGPAGPAGPAGPRGSPG 710
TR|W5NTT7|W5NTT7_SHEEP       GKGEKGETGLRGDVGSPGRDGARGAPGAVGAPGPAGANGDRGEAGPAGPAGPAGPRGSPG 710
TR|A0A1S7J1Y9|A0A1S7J1Y9_PIG GKGEKGETGLRGDVGSPGRDGARGAPGAVGAPGPAGANGDRGEAGPAGPAGPAGPRGSPG 712
SP|P02466|CO1A2_RAT          GKGEKGETGLRGEIGNPGRDGARGAPGAIGAPGPAGASGDRGEAGAAGPSGPAGPRGSPG 718
SP|P02467|CO1A2_CHICK        GKGEKGAPGLRGDTGATGRDGARGLPGAIGAPGPAGGAGDRGEGGPAGPAGPAGARGIPG 711
                             ******  ****: *  ******* ***:*******. *****.* ***:**** ** **

SP|P08123|CO1A2_HUMAN        ERGEVGPAGPNGFAGPAGAAGQPGAKGERGAKGPKGENGVVGPTGPVGAAGPAGPNGPPG 772
TR|F6RTI8|F6RTI8_HORSE       ERGEVGPAGPNGFAGPAGAAGQPGAKGERGTKGPKGENGPVGPTGPVGAAGPSGPNGPPG 770
SP|P02465|CO1A2_BOVIN        ERGEVGPAGPNGFAGPAGAAGQPGAKGERGTKGPKGENGPVGPTGPVGAAGPSGPNGPPG 770
TR|W5NTT7|W5NTT7_SHEEP       ERGEVGPAGPNGFAGPAGAAGQPGAKGERGTKGPKGENGPVGPTGPVGAAGPSGPNGPPG 770
TR|A0A1S7J1Y9|A0A1S7J1Y9_PIG ERGEVGPAGPNGFAGPAGAAGQPGAKGERGTKGPKGENGPVGPTGPVGAAGPAGPNGPPG 772
SP|P02466|CO1A2_RAT          ERGEVGPAGPNGFAGPAGSAGQPGAKGEKGTKGPKGENGIVGPTGPVGAAGPSGPNGPPG 778
SP|P02467|CO1A2_CHICK        ERGEPGPVGPSGFAGPPGAAGQPGAKGERGPKGPKGETGPTGAIGPIGASGPPGPVGAAG 771
                             **** **.**.***** *:*********:* ******.* .*  **:**:** ** *  *

SP|P08123|CO1A2_HUMAN        PAGSRGDGGPPGMTGFPGAAGRTGPPGPSGISGPPGPPGPAGKEGLRGPRGDQGPVGRTG 832
TR|F6RTI8|F6RTI8_HORSE       PAGSRGDGGPPGVTGFPGAAGRTGPPGPSGISGPPGPPGAAGKEGLRGPRGDQGPVGRAG 830
SP|P02465|CO1A2_BOVIN        PAGSRGDGGPPGATGFPGAAGRTGPPGPSGISGPPGPPGPAGKEGLRGPRGDQGPVGRSG 830
TR|W5NTT7|W5NTT7_SHEEP       PAGSRGDGGPPGATGFPGAAGRTGPPGPAGISGPPGPPGPAGKEGLRGPRGDQGPVGRTG 830
TR|A0A1S7J1Y9|A0A1S7J1Y9_PIG PAGSRGDGGPPGATGFPGAAGRIGPPGPSGISGPPGPPGPAGKEGLRGPRGDQGPVGRTG 832
SP|P02466|CO1A2_RAT          PAGSRGDGGPPGMTGFPGAAGRTGPPGPSGITGPPGPPGAAGKEGIRGPRGDQGPVGRTG 838
SP|P02467|CO1A2_CHICK        PAGPRGDAGPPGMTGFPGAAGRVGPPGPAGITGPPGPPGPAGKDGPRGLRGDVGPVGRTG 831
                             *** ***.**** ********* *****:**:******* ***:* ** *** *****:*

SP|P08123|CO1A2_HUMAN        EVGAVGPPGFAGEKGPSGEAGTAGPPGTPGPQGLLGAPGILGLPGSRGERGLPGVAGAVG 892
TR|F6RTI8|F6RTI8_HORSE       ETGASGPPGFAGEKGPSGEPGTAGPPGTPGPQGLLGAPGILGLPGSRGERGLPGVAGSLG 890
SP|P02465|CO1A2_BOVIN        ETGASGPPGFVGEKGPSGEPGTAGPPGTPGPQGLLGAPGFLGLPGSRGERGLPGVAGSVG 890
TR|W5NTT7|W5NTT7_SHEEP       EPGAAGPPGFVGEKGPSGEPGTAGPPGTPGPQGLLGAPGFLGLPGSRGERGLPGVAGSVG 890
TR|A0A1S7J1Y9|A0A1S7J1Y9_PIG ETGASGPPGFAGEKGPSGEPGTAGPPGTPGPQGILGAPGFLGLPGSRGERGLPGVAGSVG 892
SP|P02466|CO1A2_RAT          EIGASGPPGFAGEKGPSGEPGTTGPPGTAGPQGLLGAPGILGLPGSRGERGQPGIAGALG 898
SP|P02467|CO1A2_CHICK        EQGIAGPPGFAGEKGPSGEAGAAGPPGTPGPQGILGAPGILGLPGSRGERGLPGIAGATG 891
                             * *  *****.******** *::***** ****:*****:*********** **:**: *

SP|P08123|CO1A2_HUMAN        EPGPLGIAGPPGARGPPGAVGSPGVNGAPGEAGRDGNPGNDGPPGRDGQPGHKGERGYPG 952
TR|F6RTI8|F6RTI8_HORSE       EPGPLGIAGPPGARGPPGAVGAPGVNGAPGEAGRDGNPGSDGPPGRDGQPGHKGERGYPG 950
SP|P02465|CO1A2_BOVIN        EPGPLGIAGPPGARGPPGNVGNPGVNGAPGEAGRDGNPGNDGPPGRDGQPGHKGERGYPG 950
TR|W5NTT7|W5NTT7_SHEEP       EPGPLGIAGPPGARGPPGNVGNPGVNGAPGEAGRDGNPGNDGPPGRDGQPGHKGERGYPG 950
TR|A0A1S7J1Y9|A0A1S7J1Y9_PIG EPGPLGIAGPPGARGPPGAVGNPGVNGAPGEAGRDGNPGSDGPPGRDGQAGHKGERGYPG 952
SP|P02466|CO1A2_RAT          EPGPLGIAGPPGARGPPGAVGSPGVNGAPGEAGRDGNPGSDGPPGRDGQPGHKGERGYPG 958
SP|P02467|CO1A2_CHICK        EPGPLGVSGPPGARGPSGPVGSPGPNGAPGEAGRDGNPGNDGPPGRDGAPGFKGERGAPG 951
                             ******::******** * ** ** **************.********  *.***** **
```

```
SP|P08123|CO1A2_HUMAN        NIGPVGAAGAPGPHGPVGPAGKHGNRGETGPSGPVGPAGAVGPRGPSGPQGIRGDKGEPG 1012
TR|F6RTI8|F6RTI8_HORSE       NAGPVGAVGAPGPHGPVGPTGKHGHRGEPGPVGSVGPVGAVGPRGPSGPQGVRGDKGEPG 1010
SP|P02465|CO1A2_BOVIN        NAGPVGAAGAPGPQGPVGPVGKHGNRGEPGPAGAVGPAGAVGPRGPSGPQGIRGDKGEPG 1010
TR|W5NTT7|W5NTT7_SHEEP       NAGPVGAAGAPGPQGPVGPTGKHGSRGEPGPVGAVGPAGAVGPRGPSGPQGIRGDKGEPG 1010
TR|A0A1S7J1Y9|A0A1S7J1Y9_PIG NPGPAGAAGAPGPQGAVGPAGKHGNRGEPGPAGSVGPAGAVGPRGPSGPQGIRGEKGEPG 1012
SP|P02466|CO1A2_RAT          NIGPTGAAGAPGPHGSVGPAGKHGNRGEPGPAGSVGPVGAVGPRGPSGPQGIRGDKGEPG 1018
SP|P02467|CO1A2_CHICK        NPGPSGALGAPGPHGQVGPSGKPGNRGDPGPVGPVGPAGAFGPRGLAGPQGPRGEKGEPG 1011
                             * ** ** *****:* *** ** * **: ** * ***.**.**** :**** **:*****
SP|P08123|CO1A2_HUMAN        EKGPRGLPGLKGHNGLQGLPGIAGHHGDQGAPGSVGPAGPRGPAGPSGPAGKDGRTGHPG 1072
TR|F6RTI8|F6RTI8_HORSE       DKGPRGLPGIKGHNGLQGLPGLAGQHGDQGAPGSVGPAGPRGPAGPTGPVGKDGRSGQPG 1070
SP|P02465|CO1A2_BOVIN        DKGPRGLPGLKGHNGLQGLPGLAGHHGDQGAPGAVGPAGPRGPAGPSGPAGKDGRIGQPG 1070
TR|W5NTT7|W5NTT7_SHEEP       DKGPRGLPGLKGHNGLQGLPGLAGHHGDQGAPGAVGPAGPRGPAGPTGPAGKDGRTGQPG 1070
TR|A0A1S7J1Y9|A0A1S7J1Y9_PIG DKGPRGLPGLKGHNGLQGLPGLAGHHGDQGAPGPVGPAGPRGPAGPSGPAGKDGRTGQPG 1072
SP|P02466|CO1A2_RAT          DKGARGLPGLKGHNGLQGLPGLAGLHGDQGAPGPVGPAGPRGPAGPSGPIGKDGRSGHPG 1078
SP|P02467|CO1A2_CHICK        DKGHRGLPGLKGHNGLQGLPGLAGQHGDQGPPGNNGPAGPRGPPGPSGPPGKDGRNGLPG 1071
                             :** *****:***********:** ***** **  ******** **:** ***** * **
SP|P08123|CO1A2_HUMAN        TVGPAGIRGPQGHQGPAGPPGPPGPPGPPGVSGGGYDFGYDGDFYRADQPRSAPSLRPKD 1132
TR|F6RTI8|F6RTI8_HORSE       TVGPAGVRGSQGSQGPAGPPGPPGPPGPPGPSGGGYDFGYDGDFYRADQPRSPPSLRPKD 1130
SP|P02465|CO1A2_BOVIN        AVGPAGIRGSQGSQGPAGPPGPPGPPGPPGPSGGGYEFGFDGDFYRADQPRSPTSLRPKD 1130
TR|W5NTT7|W5NTT7_SHEEP       AVGPAGIRGSQGSQGPAGPPGPPGPPGPPGPSGGGYDFGFDGDFYRADQPRSPASLRPKD 1130
TR|A0A1S7J1Y9|A0A1S7J1Y9_PIG AVGPAGIRGSQGSQGPAGPPGPPGPPGPPGPSGGGYDFGYEGDFYRADQPRSPPSLRPKD 1132
SP|P02466|CO1A2_RAT          PVGPAGVRGSQGSQGPAGPPGPPGPPGPPGVSGGGYDFGFEGGFYRADQPRSQPSLRPKD 1138
SP|P02467|CO1A2_CHICK        PIGPAGVRGSHGSQGPAGPPGPPGPPGPPGPNGGGYEVGFDAEYYRADQP----SLRPKD 1127
                              :****:** :* ***************** .****:.*::. :******    ******
SP|P08123|CO1A2_HUMAN        YEVDATLKSLNNQIETLLTPEGSRKNPARTCRDLRLSHPEWSSGYYWIDPNQGCTMDAIK 1192
TR|F6RTI8|F6RTI8_HORSE       YEVDATLKSLNNQIETLLTPEGSRKNPARTCRDLRLSHPEWSSGYYWIDPNQGCTMDAIK 1190
SP|P02465|CO1A2_BOVIN        YEVDATLKSLNNQIETLLTPEGSRKNPARTCRDLRLSHPEWSSGYYWIDPNQGCTMDAIK 1190
TR|W5NTT7|W5NTT7_SHEEP       YEVDATLKSLNNQIETLLTPEGSRKNPARTCRDLRLSHPEWSSGYYWIDPNQGCTMDAIK 1190
TR|A0A1S7J1Y9|A0A1S7J1Y9_PIG YEVDATLKSLNNQIETLLTPEGSRKNPARTCRDLRLSHPEWSSGYYWIDPNQGCTMDAIK 1192
SP|P02466|CO1A2_RAT          YEVDATLKSLNNQIETLLTPEGSRKNPARTCRDLRLSHPEWKSDYYWIDPNQGCTMDAIK 1198
SP|P02467|CO1A2_CHICK        YEVDATLKTLNNQIETLLTPEGSKKNPARTCRDLRLSHPEWSSGFYWIDPNQGCTADAIR 1187
                             ********:*************:*****************.*.:********** ***:
SP|P08123|CO1A2_HUMAN        VYCDFSTGETCIRAQPENIPAKNWYRS--SKDKKHVWLGETINAGSQFEYNVEGVTSKEM 1250
TR|F6RTI8|F6RTI8_HORSE       VYCDFSTGETCIRAQPENIPAKNWYRS--SKAKKHIWLGETINGGTQFEYNVEGVTTKEM 1248
SP|P02465|CO1A2_BOVIN        VYCDFSTGETCIRAQPEDIPVKNWYRN--SKAKKHVWVGETINGGTQFEYNVEGVTTKEM 1248
TR|W5NTT7|W5NTT7_SHEEP       VYCDFSTGETCIRAQPEDIPVKNWYRN--SKAKKHVWVGETINGGTQFEYNVEGVTTKEM 1248
TR|A0A1S7J1Y9|A0A1S7J1Y9_PIG VYCDFSTGETCIRAQPENIPAKNWYRN--SKVKKHVWLGETINGGTQFEYNMEGVTTKEM 1250
SP|P02466|CO1A2_RAT          VYCDFSTGETCIQAQPVNTPAKNAYSR--AQANKHVWLGETINGGSQFEYNAEGVSSKEM 1256
SP|P02467|CO1A2_CHICK        AYCDFATGETCIHASLEDIPTKTWYVSKNPKDKKHIWFGETINGGTQFEYNGEGVTTKDM 1247
                             .****:******:*.  : *.*. *      : :**:*.*****.*:***** ***::*:*
SP|P08123|CO1A2_HUMAN        ATQLAFMRLLANYASQNITYHCKNSIAYMDEETGNLKKAVILQGSNDVELVAEGNSRFTY 1310
TR|F6RTI8|F6RTI8_HORSE       ATQLAFMRLLANHASQNITYHCKNSIAYLDEETGNLKKAVTLQGSNDVELVAEGNSRFTY 1308
SP|P02465|CO1A2_BOVIN        ATQLAFMRLLANHASQNITYHCKNSIAYMDEETGNLKKAVILQGSNDVELVAEGNSRFTY 1308
TR|W5NTT7|W5NTT7_SHEEP       ATQLAFMRLLANHASQNITYHCKNSIAYMDEETGNLKKAVILQGSNDVELVAEGNSRFTY 1308
```

```
TR|A0A1S7J1Y9|A0A1S7J1Y9_PIG ATQLAFMRLLANHASQNITYHCKNSIAYMDEETGNLKKAVILQGSNDVELVAEGNSRFTY 1310
SP|P02466|CO1A2_RAT          ATQLAFMRLLANRASQNITYHCKNSIAYLDEETGRLNKAVILQGSNDVELVAEGNSRFTY 1316
SP|P02467|CO1A2_CHICK        ATQLAFMRLLANHASQNITYHCKNSIAYMDEETGNLKKAVILQGSNDVELRAEGNSRFTF 1307
                             ************ ***************:*****.*:*** ********* ********:
SP|P08123|CO1A2_HUMAN        TVLVDGCSKKTNEWGKTIIEYKTNKPSRLPFLDIAPLDIGGADQEFFVDIGPVCFK 1366
TR|F6RTI8|F6RTI8_HORSE       TVLVDGCSRKTNEWGKTIIEYKTNKPSRLPILDIALLDIGGADQEFGLDIGPVCFK 1364
SP|P02465|CO1A2_BOVIN        TVLVDGCSKKTNEWQKTIIEYKTNKPSRLPILDIAPLDIGGADQEIRLNIGPVCFK 1364
TR|W5NTT7|W5NTT7_SHEEP       TVLVDGCSKKTNEWKKTIIEYKTNKPSRLPILDIAPLDIGGADQEIRLNIGPVCFK 1364
TR|A0A1S7J1Y9|A0A1S7J1Y9_PIG TVLVDGCSKKTNEWRKTIIEYKTNKPSRLPILDIAPLDIGDADQEVSVDVGPVCFK 1366
SP|P02466|CO1A2_RAT          TVLVDGCSKKTNEWDKTVIEYKTNKPSRLPFLDIAPLDIGGTNQEFRVEVGPVCFK 1372
SP|P02467|CO1A2_CHICK        SVLVDGCSKKNNKWGKTIIEYRTNKPSRLPILDIAPLDIGGADQEFGLHIGPVCFK 1363
                             :*******:*.*:* **:***:********:**** ****.::**. :.:******
```

© 2020 by the authors. Submitted for possible open access publication under the terms and conditions of the Creative Commons Attribution (CC BY) license (http://creativecommons.org/licenses/by/4.0/).
